# Trapezoidal-Type Inequalities for Strongly Convex and Quasi-Convex Functions via Post-Quantum Calculus

**DOI:** 10.3390/e23101238

**Published:** 2021-09-22

**Authors:** Humaira Kalsoom, Miguel Vivas-Cortez, Muhammad Amer Latif

**Affiliations:** 1 Department of Mathematical, Zhejiang Normal University, Jinhua 321004, China; humaira87@zju.edu.cn; 2 Escuela de Ciencias Físicas y Matemáticas, Facultad de Ciencias Naturales y Exactas Pontificia Universidad Católica del Ecuador, Sede Quito 17-01-2184, Ecuador; 3 Department of Basic Sciences, Deanship of Preparatory Year, King Faisal University, Hofuf 31982, Al-Hasa, Saudi Arabia

**Keywords:** *(p,q)*-calculus, trapezoidal *(p,q)*_*κ*1_-integral and *(p,q)*^*κ*2^-integral, strongly convex functions, strongly quasi-convex functions

## Abstract

In this paper, we establish new $\left(p,q\right){}_{\kappa_{1}}$-integral and $\left(p,q\right){}^{\kappa_{2}}$-integral identities. By employing these new identities, we establish new $\left(p,q\right){}_{\kappa_{1}}$ and $\left(p,q\right){}^{\kappa_{2}}$- trapezoidal integral-type inequalities through strongly convex and quasi-convex functions. Finally, some examples are given to illustrate the investigated results.

## 1. Introduction and Preliminaries

Quantum calculus, often known as *q*-calculus, is a branch of mathematics that studies calculus without limits. Euler’s work on infinite series, which he integrated with Newton’s work on parameters, served as the idea for the q-calculus analysis, which was founded in the eighteenth century by famous mathematician Leonhard Euler (1707–1783). In 1910, F. H. Jackson [1] used L. Euler’s expertise to define the q-derivative and *q*-integral of any function on the interval (0,∞) using the *q*-calculus of infinite series. Quantum calculus has a very long history. However, to keep up with the times, it has undergone rapid growth over the past few decades. However, in order to stay current, it has experienced tremendous development over the last several decades. I am a strong believer in this as it serves as a link between mathematics and physics, which is useful when working with physics. To get more information, please check the application and results of Ernst [2], Gauchman [3], and Kac and Cheung [4] in the theory of quantum calculus and theory of inequalities in quantum calculus. In previous papers, the authors Ntouyas and Tariboon [5] investigated how quantum-derivatives and quantum-integrals are solved over the intervals of the form $[\kappa_{1},\kappa_{2}]\subset R$. In addition, they studied the characteristics and specific results of initial value problems in impulsive *q*-differential equations of the first and second order. Furthermore, set a number of quantum analogs for some well-known effects, for example, Hölder inequality, Hermite–Hadamard inequality and Ostrowski inequality, Cauchy–Bunyakovsky–Schwarz, Gruss, Gruss–Cebysev, and other integral inequalities that use classical convexity. Furthermore, Noor et al. [6], Sudsutad et al. [7], and Zhuang et al. [8] played an active role in the study and some integral inequalities have been established which give quantum analog for the right part of Hermite–Hadamard inequality by using *q*-differentiable convex and quasi-convex functions. Numerous mathematicians have carried out research in the area of *q*-calculus analysis; interested readers may check the works in [9,10,11,12,13,14,15,16,17,18,19].

*q*-calculus generalization is post-quantum or, often, is referred to as (p,q) calculus. Post-quantum calculus is a recent advancement in the study of quantum calculus that contains *p* and *q*-numbers with two independent variables *p* and *q*. Quantum calculus is concerned with *q*-numbers with a single basis. Therefore, (p,q)-calculus is known as two-parameter quantum calculus. Applications play significant roles in mathematics and physics, such as combinatorics, fractals, special functions, number theory, dynamical systems, and mechanics. Many additional (p,q)-analogs of classical inequalities have been discovered since the publication of this article. In (p,q)-calculus, we get the *q*-calculus formula when p=1 and the original mathematical formula when $q\to 1^{-}$. Motivated by the research work going on, Tunç and Göv [20] introduced the concepts of (p,q)-derivatives and (p,q)-integrals on finite intervals. Kunt et al. [21] used (p,q)-differentiable convex and quasi-convex functions to prove the left side of the (p,q)-Hermite–Hadamard inequality, and then generated some new (p,q)-Hermite–Hadamard inequalities. Latif et al. [22] proved the new variations in trapezoidal inequalities after quantum have been shown to be achieved using the new (p,q)-integral identity. Based on (p,q)-calculus, many works have been published by many researchers, see in [23,24,25,26,27,28,29,30] for more details and the references cited therein.

Integral inequalities are a fundamental tool in both pure and applied mathematics for constructing qualitative and quantitative properties. This perspective facilitated the discovery of novel and significant findings in a wide variety of areas of the mathematical and engineering sciences and provided a comprehensive framework for the study of many issues. Numerous researchers have explored the different types of convex sets and convex functions.

Suppose that the function K:I⊆R→R is said to be convex, if K meets the following inequality:

$$K\left(\tau \kappa_{1}+\left(1-\tau \right)\kappa_{2}\right)\leq \tau K\left(\kappa_{1}\right)+\left(1-\tau \right)K\left(\kappa_{2}\right)$$

for all $\kappa_{1},\kappa_{2}\in I$ and τ∈[0,1].

Hermite–Hadamard inequalities are among the most well-known results in the theory of convex functional analysis. It has an intriguing geometric representation that is applicable to a wide variety of situations.

According to the exceptional inequality, if K:I⊆R→R is a convex mapping on the interval *I* of real numbers and $\kappa_{1},\kappa_{2}\in I$ with $\kappa_{1}<\kappa_{2}$. Then,

(1) $$K\left(\frac{\kappa_{1}+\kappa_{2}}{2}\right)\leq \frac{1}{\kappa_{2}-\kappa_{1}}\int_{\kappa_{1}}^{\kappa_{2}}K\left(\tau \right)d\tau \leq \frac{K\left(\kappa_{1}\right)+K\left(\kappa_{2}\right)}{2}.$$

Inequality (1) was introduced by C. Hermite [31] and investigated by J. Hadamard [32] in 1893. For K to be concave, both inequalities hold in the inverted direction. Many mathematicians have paid great attention to the inequality of Hermite–Hadamard due to its quality and validity in mathematical inequalities. For significant developments, modifications, and consequences regarding the Hermite–Hadamard uniqueness property and general convex function definitions, for essential details, the interested reader would like to refer to the works in [33,34,35] and references therein.

Different inequalities are used to represent convex functions. Convex functions are responsible for several well-known inequalities. Strongly convexity is a reinforcement of the concept of convexity; some aspects of strongly convex functions are just “stronger versions” of known convex properties. Polyak [36] introduced the strongly convex function as

**Definition** **1** ([36])**.** *A function K:I→R with the modulus χ≥1 is called strongly convex function, if*

$$K\left(\tau \kappa_{1}+\left(1-\tau \right)\kappa_{2}\right)\leq \tau K\left(\kappa_{1}\right)+\left(1-\tau \right)K\left(\kappa_{2}\right)-\tau \left(1-\tau \right)\chi {\left(\kappa_{2}-\kappa_{1}\right)}^{2}$$

*for all $\kappa_{1},\kappa_{2}\in I,\kappa_{1}<\kappa_{2}$ and τ∈[0,1].*

Strongly convex functions play a significant role in optimization, mathematical economics, nonlinear programming, etc. Some properties of strongly convex functions are just stronger versions of properties of convex functions. Moreover, Nikodem et al. [37] introduced new characterizations of inner product spaces among normed spaces involving the notion of strong convexity.

Note that quasi-convex functions are a generalization of the convex function class, as there are quasi-convex functions that are not convex.

**Definition** **2** ([38])**.** *A function K:I→R with the modulus χ≥1 is strongly quasi-convex function, if*

$$K\left(\tau \kappa_{1}+\left(1-\tau \right)\kappa_{2}\right)\leq \max\left\{K\left(\kappa_{1}\right),K\left(\kappa_{2}\right)\right\}-\tau \left(1-\tau \right)\chi {\left(\kappa_{2}-\kappa_{1}\right)}^{2}$$

*for all $\kappa_{1},\kappa_{2}\in I,\kappa_{1}<\kappa_{2}$ and τ∈[0,1].*

**Remark** **1.** *The notion of strongly quasi-convexity strengthens the concept of quasi-convexity.*

Latif et al. [22] proved quantum estimates of (p,q)-trapezoidal integral inequalities through convex and quasi-convex functions

**Theorem** **1** ([22])**.** *Suppose that $K:\left[\kappa_{1},\kappa_{2}\right]\to R$ is a ${\left(p,q\right)}_{\kappa_{1}}$-differentiable function on $\left(\kappa_{1},\kappa_{2}\right)$, ${}_{\kappa_{1}}D_{p,q}K$ is a ${\left(p,q\right)}_{\kappa_{1}}$-integrable on $\left[\kappa_{1},\kappa_{2}\right]$ and 0<q<p≤1. If ${\left|{}_{\kappa_{1}}D_{p,q}K\right|}^{\sigma }$ is a convex functions on $\left[\kappa_{1},\kappa_{2}\right]$ with σ≥1, then*

(2) $$\begin{array}{c} |\frac{1}{p\left(\kappa_{2}-\kappa_{1}\right)}\int_{\kappa_{1}}^{\left(1-p\right)\kappa_{1}+p\kappa_{2}}K\left(x\right){\;}_{\kappa_{1}}d_{p,q}x-\frac{qK\left(\kappa_{1}\right)+pK\left(\kappa_{2}\right)}{{\left[2\right]}_{p,q}}|\leq \frac{q\left(\kappa_{2}-\kappa_{1}\right)}{{\left[2\right]}_{p,q}}{\left[T_{1}\left(p,q\right)\right]}^{1-\frac{1}{\sigma }}\\ \times {\left[T_{2}\left(p,q\right){\left|{}_{\kappa_{1}}D_{p,q}K\left(\kappa_{1}\right)\right|}^{\sigma }+T_{3}\left(p,q\right){\left|{}_{\kappa_{1}}D_{p,q}K\left(\kappa_{2}\right)\right|}^{\sigma }\right]}^{\frac{1}{\sigma }}, \end{array}$$

*where*

$$\begin{array}{c} T_{1}\left(p,q\right)=\frac{2\left({\left[2\right]}_{p,q}-1\right)}{{\left[2\right]}_{p,q}^{2}}\\ T_{2}\left(p,q\right)=\frac{q\left[\left(5p^{3}-4p^{2}-2p+2\right)+\left(6p^{2}-4p-2\right)q+\left(5p-2\right)q^{2}+2q^{3}\right]+\left(2p^{4}-2p^{3}-2p^{2}+2p\right)}{{\left[2\right]}_{p,q}^{3}{\left[3\right]}_{p,q}}\\ T_{3}\left(p,q\right)=\frac{q\left[\left(p^{3}-2+2p\right)+\left(2p^{2}+2\right)q+pq^{2}\right]+2p^{2}-2p}{{\left[2\right]}_{p,q}^{3}{\left[3\right]}_{p,q}}. \end{array}$$

**Theorem** **2** ([22])**.** *Suppose that $K:\left[\kappa_{1},\kappa_{2}\right]\to R$ is a ${\left(p,q\right)}_{\kappa_{1}}$-differentiable function on $\left(\kappa_{1},\kappa_{2}\right)$, ${}_{\kappa_{1}}D_{p,q}K$ is a ${\left(p,q\right)}_{\kappa_{1}}$-integrable on $\left[\kappa_{1},\kappa_{2}\right]$ and 0<q<p≤1. If ${\left|{}_{\kappa_{1}}D_{p,q}K\right|}^{\sigma }$ is a quasi-convex functions on $\left[\kappa_{1},\kappa_{2}\right]$ with σ≥1, then*

(3) $$\begin{array}{c} \left|\frac{1}{p\left(\kappa_{2}-\kappa_{1}\right)}\int_{\kappa_{1}}^{\left(1-p\right)\kappa_{1}+p\kappa_{2}}K\left(x\right){\;}_{\kappa_{1}}d_{p,q}x-\frac{qK\left(\kappa_{1}\right)+pK\left(\kappa_{2}\right)}{{\left[2\right]}_{p,q}}\right|\\ \leq \frac{q\left(\kappa_{2}-\kappa_{1}\right)}{{\left[2\right]}_{p,q}}\left[T_{1}\left(p,q\right)\right]\max{\left[{\left|{}_{\kappa_{1}}D_{p,q}K\left(\kappa_{1}\right)\right|}^{\sigma },{\left|{}_{\kappa_{1}}D_{p,q}K\left(\kappa_{2}\right)\right|}^{\sigma }\right]}^{\frac{1}{\sigma }}, \end{array}$$

*where*

$$T_{1}\left(p,q\right)=\frac{2\left({\left[2\right]}_{p,q}-1\right)}{{\left[2\right]}_{p,q}^{2}}.$$

Several fundamental inequalities that are well known in classical analysis, like Hölder inequality, Ostrowski inequality, Cauchy–Schwarz inequality, Grüess–Chebyshev inequality, and Grüess inequality. Using classical convexity, other fundamental inequalities have been proven and applied to *q*-calculus.

Our objective is to develop improved trapezoidal type inequalities by using post-quantum calculus and to support this claim graphically.

### 1.1. q-Derivatives and Integrals

In this section, we discuss some required definitions of *q*, (p,q)-Calculus and important quantum integral inequalities for Hermite–Hadamard on left and right sides bonds. Throughout this paper, we are using constants 0<q<1 and 0<q<p≤1.

The ${\left[m\right]}_{q}$ integers are known as *q*-integers and are written as

$${\left[m\right]}_{q}=1+q+q^{2}\cdots q^{m}-1=\frac{1-q^{m}}{1-q},\;for\;m=1,2\ldots$$

$${\left[m\right]}_{q}=m,\;for\;m=1.$$

The ${\left[m\right]}_{q}!$ and ${\left[\begin{array}{c} m\\ i \end{array}\right]}_{q}!$ are denoted as *q*-factorial and *q*-binomial, respectively, and are written as follows:

$$\begin{array}{cc} {\left[m\right]}_{q}! & =\prod_{i=1}^{m}{\left[i\right]}_{q},\;m\geq 1,\;{\left[0\right]}_{q}!=1,\\ {\left[\begin{array}{c} m\\ i \end{array}\right]}_{q}! & =\frac{{\left[m\right]}_{q}!}{{\left[m-i\right]}_{q}!{\left[i\right]}_{q}!}. \end{array}$$

In the early twentieth century, the Reverend Frank Hilton Jackson made major contributions to the classical concept of a derivative of a function at a point, which allowed for a more straightforward study of ordinary calculus and number theory in these investigations. Jackson is responsible for numerous seminal studies in the subject, including that in [1], in addition to creating the *q*-analogs of certain major results discovered in these disciplines.

(4) $$D_{q}K\left(\kappa \right)=\frac{K\left(\kappa \right)-K\left(q\kappa \right)}{\left(1-q\right)\kappa },\;\kappa \neq 0.$$

The classic Jackson approach is given below.

(5) $$\int_{0}^{\kappa_{2}}K\left(\kappa \right)\;d_{q}\kappa =\left(1-q\right)\kappa_{2}\sum_{n=0}^{\infty }q^{n}K\left(\kappa_{2}q^{n}\right),$$

provided the sum converge absolutely.

The *q*-Jackson integral in a generic interval $\left[\kappa_{1},\kappa_{2}\right]$ is defined as follows:

$$\int_{\kappa_{1}}^{\kappa_{2}}K\left(\kappa \right)d_{q}\kappa =\int_{0}^{\kappa_{2}}K\left(\kappa \right)d_{q}\kappa -\int_{0}^{\kappa_{1}}K\left(\kappa \right)d_{q}\kappa .$$

Whenever q approaches 1, the number theory, deduction, and ordinary integration findings become polynomial expressions in a real variable *q*.

**Definition** **3** ([5])**.** *We suppose that $K:\left[\kappa_{1},\kappa_{2}\right]\to R$ be an arbitrary function. Then $q_{\kappa_{1}}$-derivative of K at $\kappa \in \left[\kappa_{1},\kappa_{2}\right]$ is defined as follows:*

(6) $${}_{\kappa_{1}}D_{q}K\left(\kappa \right)=\frac{K\left(\kappa \right)-K\left(q\kappa +\left(1-q\right)\kappa_{1}\right)}{\left(1-q\right)\left(\kappa -\kappa_{1}\right)},\;\kappa \neq \kappa_{1}.$$

*As K is an arbitrary function from $\left[\kappa_{1},\kappa_{2}\right]$ to R, so for $\kappa =\kappa_{1}$, we define ${}_{\kappa_{1}}D_{q}K\left(\kappa_{1}\right)=\lim_{\kappa \to \kappa_{1}}{}_{\kappa_{1}}D_{q}K\left(\kappa \right)$. The function K is called $q_{\kappa_{1}}$-differentiable on $\left[\kappa_{1},\kappa_{2}\right]$, if ${}_{\kappa_{1}}D_{q}K\left(\kappa \right)$ exists for all $\kappa \in \left[\kappa_{1},\kappa_{2}\right]$.*

**Remark** **2.** *Note that if $\kappa_{1}=0$ in (6), then we obtain the similar q-derivative that is defined in (4).*

The following lemma is play key part to calculate $q_{\kappa_{1}}$-derivatives.

**Lemma** **1** ([5])**.** *Taking ξ∈R, we have*

$${}_{\kappa_{1}}D_{q}{\left(x-\kappa_{1}\right)}^{\xi }=\left(\frac{1-q^{\xi }}{1-q}\right){\left(x-\kappa_{1}\right)}^{\xi -1}.$$

**Definition** **4** ([5])**.** *We suppose that $K:\left[\kappa_{1},\kappa_{2}\right]\to R$ be an arbitrary function, then the $q_{\kappa_{1}}$-definite integral on $\left[\kappa_{1},\kappa_{2}\right]$ is described as below*

(7) $$\int_{\kappa_{1}}^{\kappa }K\left(\kappa \right){\;}_{\kappa_{1}}d_{q}\kappa =\left(1-q\right)\left(\kappa -\kappa_{1}\right)\sum_{n=0}^{\infty }q^{n}K\left(q^{n}\kappa +\left(1-q^{n}\right)\kappa_{1}\right),\;\kappa \in \left[\kappa_{1},\kappa_{2}\right].$$

The following properties are very important in quantum calculus:

**Theorem** **3** ([5])**.** *Let K:I→R be a continuous function. Then,*

*1.*  *${}_{\kappa_{1}}D_{q}\int_{\kappa_{1}}^{x}K\left(\tau \right){\;}_{\kappa_{1}}d_{q}\tau =K\left(x\right)$;*
*2.*  *$\int_{\chi }^{x}{}_{\kappa_{1}}D_{q}K\left(\tau \right){\;}_{\kappa_{1}}d_{q}\tau =K\left(x\right)-K\left(\chi \right)$, $\chi \in \left(\kappa_{1},x\right).$*

The following is useful results for evaluating such $q\kappa_{1}$-integrals.

**Lemma** **2** ([5])**.** *The following formula holds for $\zeta \in R\setminus \left\{-1\right\}$, then*

$$\int_{\kappa_{1}}^{\sigma }{\left(\tau -\kappa_{1}\right)}^{\zeta }{\;}_{\kappa_{1}}d_{q}\tau =\left(\frac{1-q}{1-q^{\zeta +1}}\right){\left(\sigma -\kappa_{1}\right)}^{\zeta +1}.$$

In [9], Alp et al. established the $q_{\kappa_{1}}$-Hermite–Hadamard inequalities for convexity, which is defined as follows:

**Theorem** **4** ([9])**.** *We suppose that $K:\left[\kappa_{1},\kappa_{2}\right]\to R$ is a convex differentiable function on $\left[\kappa_{1},\kappa_{2}\right]$. Then $q_{\kappa_{1}}$-Hermite–Hadamard inequalities are as follows:*

(8) $$K\left(\frac{q\kappa_{1}+\kappa_{2}}{{\left[2\right]}_{q}}\right)\leq \frac{1}{\kappa_{2}-\kappa_{1}}\int_{\kappa_{1}}^{\kappa_{2}}K\left(\kappa \right){\;}_{\kappa_{1}}d_{q}\kappa \leq \frac{qK\left(\kappa_{1}\right)+K\left(\kappa_{2}\right)}{{\left[2\right]}_{q}}.$$

On the other hand, the following new description of $q^{\kappa_{2}}$-derivative, $q^{\kappa_{2}}$-integration and related $q^{\kappa_{2}}$-Hermite–Hadamard form inequalities were given by Bermudo et al. [15]

**Definition** **5** ([15])**.** *We suppose that $K:\left[\kappa_{1},\kappa_{2}\right]\to R$ is an arbitrary function, then $q^{\kappa_{2}}$-derivative of K at $\kappa \in \left[\kappa_{1},\kappa_{2}\right]$ is defined as follows:*

$${}^{\kappa_{2}}D_{q}K\left(\kappa \right)=\frac{K\left(q\kappa +\left(1-q\right)\kappa_{2}\right)-K\left(\kappa \right)}{\left(1-q\right)\left(\kappa_{2}-\kappa \right)},\;\kappa \neq \kappa_{2}.$$

As K is an arbitrary function from $\left[\kappa_{1},\kappa_{2}\right]$ to R, so for $\kappa =\kappa_{2}$, we define ${}^{\kappa_{2}}D_{q}K\left(\kappa_{2}\right)=\lim_{\kappa \to \kappa_{2}}{}^{\kappa_{2}}D_{q}K\left(\kappa \right)$. The function K is called $q^{\kappa_{2}}$-differentiable on $\left[\kappa_{1},\kappa_{2}\right]$, if ${}^{\kappa_{2}}D_{q}K\left(\kappa \right)$ exists for all $\kappa \in \left[\kappa_{1},\kappa_{2}\right]$.

**Definition** **6** ([15])**.** *We suppose that $K:\left[\kappa_{1},\kappa_{2}\right]\to R$ is an arbitrary function. Then, the $q^{\kappa_{2}}$-definite integral on $\left[\kappa_{1},\kappa_{2}\right]$ is defined as*

$$\int_{\kappa }^{\kappa_{2}}K\left(\kappa \right){\;}^{\kappa_{2}}d_{q}\kappa =\left(1-q\right)\left(\kappa_{2}-\kappa \right)\sum_{n=0}^{\infty }q^{n}K\left(q^{n}\kappa +\left(1-q^{n}\right)\kappa_{2}\right),\;\kappa \in \left[\kappa_{1},\kappa_{2}\right].$$

**Theorem** **5** ([15])**.** *We suppose that $K:\left[\kappa_{1},\kappa_{2}\right]\to R$ be a convex function on $\left[\kappa_{1},\kappa_{2}\right].$ Then, $q^{\kappa_{2}}$-Hermite–Hadamard inequalities are as follows:*

(9) $$K\left(\frac{\kappa_{1}+q\kappa_{2}}{{\left[2\right]}_{q}}\right)\leq \frac{1}{\kappa_{2}-\kappa_{1}}\int_{\kappa_{1}}^{\kappa_{2}}K\left(\kappa \right){\;}^{\kappa_{2}}d_{q}\kappa \leq \frac{K\left(\kappa_{1}\right)+qK\left(\kappa_{2}\right)}{{\left[2\right]}_{q}}.$$

From Theorems 4 and 5, one can the following inequalities:

**Corollary** **1** ([15])**.** *For any convex function $K:\left[\kappa_{1},\kappa_{2}\right]\to R,$ we have*

(10) $$K\left(\frac{q\kappa_{1}+\kappa_{2}}{{\left[2\right]}_{q}}\right)+K\left(\frac{\kappa_{1}+q\kappa_{2}}{{\left[2\right]}_{q}}\right)\leq \frac{1}{\kappa_{2}-\kappa_{1}}\left\{\int_{\kappa_{1}}^{\kappa_{2}}K\left(\kappa \right){\;}_{\kappa_{1}}d_{q}\kappa +\int_{\kappa_{1}}^{\kappa_{2}}K\left(\kappa \right){\;}^{\kappa_{2}}d_{q}\kappa \right\}\leq K\left(\kappa_{1}\right)+K\left(\kappa_{2}\right)$$

*and*

(11) $$K\left(\frac{\kappa_{1}+\kappa_{2}}{2}\right)\leq \frac{1}{2\left(\kappa_{2}-\kappa_{1}\right)}\left\{\int_{\kappa_{1}}^{\kappa_{2}}K\left(\kappa \right){\;}_{\kappa_{1}}d_{q}\kappa +\int_{\kappa_{1}}^{\kappa_{2}}K\left(\kappa \right){\;}^{\kappa_{2}}d_{q}\kappa \right\}\leq \frac{K\left(\kappa_{1}\right)+K\left(\kappa_{2}\right)}{2}.$$

### 1.2. (p,q)-Derivatives and Integrals

In this section, we review some fundamental notions and symbols of $\left(p,q\right)$-calculus.

The ${\left[m\right]}_{p,q}$ integers are known as (p,q) integers and are written as

$${\left[m\right]}_{p,q}=\frac{p^{m}-q^{m}}{p-q}.$$

The ${\left[m\right]}_{p,q}!$ and ${\left[\begin{array}{c} m\\ i \end{array}\right]}_{p,q}!$ are denoted as (p,q)-factorial and (p,q)-binomial, respectively, and are written as follows:

$$\begin{array}{cc} {\left[m\right]}_{p,q}! & =\prod_{i=1}^{m}{\left[i\right]}_{p,q},\;m\geq 1,\;{\left[0\right]}_{p,q}!=1,\\ {\left[\begin{array}{c} m\\ i \end{array}\right]}_{p,q}! & =\frac{{\left[m\right]}_{p,q}!}{{\left[m-i\right]}_{p,q}!{\left[i\right]}_{p,q}!}. \end{array}$$

**Definition** **7** ([20])**.** *The $\left(p,q\right)$-derivative of mapping $K:\left[\kappa_{1},\kappa_{2}\right]\to R$ is given as*

(12) $$D_{p,q}K\left(\kappa \right)=\frac{K\left(p\kappa \right)-K\left(q\kappa \right)}{\left(p-q\right)\kappa },\;\kappa \neq 0.$$

**Definition** **8** ([20])**.** *The ${\left(p,q\right)}_{\kappa_{1}}$-derivative of mapping $K:\left[\kappa_{1},\kappa_{2}\right]\to R$ is given as*

(13) $${}_{\kappa_{1}}D_{p,q}K\left(\kappa \right)=\frac{K\left(p\kappa +\left(1-p\right)\kappa_{1}\right)-K\left(q\kappa +\left(1-q\right)\kappa_{1}\right)}{\left(p-q\right)\left(\kappa -\kappa_{1}\right)},\;\kappa \neq \kappa_{1}.$$

As K is an arbitrary function from $\left[\kappa_{1},\kappa_{2}\right]$ to R, so for $\kappa =\kappa_{1}$, we define ${}_{\kappa_{1}}D_{p,q}K\left(\kappa_{1}\right)=\lim_{\kappa \to \kappa_{1}}{}_{\kappa_{1}}D_{p,q}K\left(\kappa \right)$. The function K is called ${\left(p,q\right)}_{\kappa_{1}}$-differentiable on $\left[\kappa_{1},\kappa_{2}\right]$, if ${}_{\kappa_{1}}D_{p,q}K\left(\kappa \right)$ exists for all $\kappa \in \left[\kappa_{1},\kappa_{2}\right]$.

**Definition** **9** ([23])**.** *The ${\left(p,q\right)}^{\kappa_{2}}$-derivative of mapping $K:\left[\kappa_{1},\kappa_{2}\right]\to R$ is given as*

(14) $${}^{\kappa_{2}}D_{p,q}K\left(\kappa \right)=\frac{K\left(q\kappa +\left(1-q\right)\kappa_{2}\right)-K\left(p\kappa +\left(1-p\right)\kappa_{2}\right)}{\left(p-q\right)\left(\kappa_{2}-x\right)},\;\kappa \neq \kappa_{2}.$$

As K is an arbitrary function from $\left[\kappa_{1},\kappa_{2}\right]$ to R, so for $\kappa =\kappa_{2}$, we define ${}^{\kappa_{2}}D_{p,q}K\left(\kappa_{2}\right)=\lim_{\kappa \to \kappa_{2}}{}^{\kappa_{2}}D_{p,q}K\left(\kappa \right)$. The function K is called ${\left(p,q\right)}^{\kappa_{2}}$-differentiable on $\left[\kappa_{1},\kappa_{2}\right]$, if ${}^{\kappa_{2}}D_{p,q}K\left(\kappa \right)$ exists for all $\kappa \in \left[\kappa_{1},\kappa_{2}\right]$.

**Definition** **10** ([20])**.** *The definite (p,q)${}_{\kappa_{1}}$-integral of mapping $K:\left[\kappa_{1},\kappa_{2}\right]\to R$ on $\left[\kappa_{1},\kappa_{2}\right]$ is stated as*

(15) $$\int_{\kappa_{1}}^{\kappa }K\left(\tau \right){\;}_{\kappa_{1}}d_{p,q}\tau =\left(p-q\right)\left(\kappa -\kappa_{1}\right)\sum_{n=0}^{\infty }\frac{q^{n}}{p^{n+1}}K\left(\frac{q^{n}}{p^{n+1}}\kappa +\left(1-\frac{q^{n}}{p^{n+1}}\right)\kappa_{1}\right).$$

**Definition** **11.** *From [23], the definite (p,q)${}^{\kappa_{2}}$-integral of mapping $K:\left[\kappa_{1},\kappa_{2}\right]\to R$ on $\left[\kappa_{1},\kappa_{2}\right]$ is stated as*

(16) $$\int_{\kappa }^{\kappa_{2}}K\left(\tau \right){\;}^{\kappa_{2}}d_{p,q}\tau =\left(p-q\right)\left(\kappa_{2}-\kappa \right)\sum_{n=0}^{\infty }\frac{q^{n}}{p^{n+1}}K\left(\frac{q^{n}}{p^{n+1}}\kappa +\left(1-\frac{q^{n}}{p^{n+1}}\right)\kappa_{2}\right).$$

**Remark** **3.** *If we take $\kappa_{1}=0$ and $\kappa =\kappa_{2}=1$ in (15), then we have*

$$\int_{0}^{1}K\left(t\right){\;}_{0}d_{p,q}t=\left(p-q\right)\sum_{n=0}^{\infty }\frac{q^{n}}{p^{n+1}}K\left(\frac{q^{n}}{p^{n+1}}\right).$$

*Similarly, by taking $\kappa =\kappa_{1}=0$ and $\kappa_{2}=1$ in (16), then we obtain that*

$$\int_{0}^{1}K\left(\tau \right){\;}^{1}d_{p,q}\tau =\left(p-q\right)\sum_{n=0}^{\infty }\frac{q^{n}}{p^{n+1}}K\left(1-\frac{q^{n}}{p^{n+1}}\right).$$

In [21], Kunt et al. proved the following Hermite–Hadamard-type inequalities for convex functions via (p,q)${}_{\kappa_{1}}$ integral:

**Theorem** **6** ([21])**.** *For a convex mapping $K:\left[\kappa_{1},\kappa_{2}\right]\to R$ which is differentiable on $\left[\kappa_{1},\kappa_{2}\right],$ the following inequalities hold for ${\left(p,q\right)}_{\kappa_{1}}$-integral:*

(17) $$K\left(\frac{q\kappa_{1}+p\kappa_{2}}{{\left[2\right]}_{p,q}}\right)\leq \frac{1}{p\left(\kappa_{2}-\kappa_{1}\right)}\int_{\kappa_{1}}^{p\kappa_{2}+\left(1-p\right)\kappa_{1}}K\left(\kappa \right){\;}_{\kappa_{1}}d_{p,q}\kappa \leq \frac{qK\left(\kappa_{1}\right)+pK\left(\kappa_{2}\right)}{{\left[2\right]}_{p,q}}.$$

**Lemma** **3.** *We have the following equalities:*

$$\int_{\kappa_{1}}^{\kappa_{2}}{\left(\kappa_{2}-\kappa \right)}^{\alpha }{\;}^{\kappa_{2}}d_{p,q}\kappa =\frac{{\left(\kappa_{2}-\kappa_{1}\right)}^{\alpha +1}}{{\left[\alpha +1\right]}_{p,q}}$$

$$\int_{\kappa_{1}}^{\kappa_{2}}{\left(\kappa -\kappa_{1}\right)}^{\alpha }{\;}_{\kappa_{1}}d_{p,q}\kappa =\frac{{\left(\kappa_{2}-\kappa_{1}\right)}^{\alpha +1}}{{\left[\alpha +1\right]}_{p,q}}$$

*where α∈R∖{−1}.*

**Proof.**  From Definition 11, we have

$$\begin{array}{cc} \int_{\kappa_{1}}^{\kappa_{2}}{\left(\kappa_{2}-\kappa \right)}^{\alpha }{\;}^{\kappa_{2}}d_{p,q}\kappa & =\left(p-q\right)\left(\kappa_{2}-\kappa_{1}\right)\sum_{n=0}^{\infty }\frac{q^{n}}{p^{n+1}}{\left(\kappa_{2}-\left(\frac{q^{n}}{p^{n+1}}\kappa_{1}+\left(1-\frac{q^{n}}{p^{n+1}}\right)\kappa_{2}\right)\right)}^{\alpha }\\ \; & =\left(p-q\right)\left(\kappa_{2}-\kappa_{1}\right)\sum_{n=0}^{\infty }\frac{q^{n}}{p^{n+1}}{\left(\frac{q^{n}}{p^{n+1}}\left(\kappa_{2}-\kappa_{1}\right)\right)}^{\alpha }\\ \; & =\left(p-q\right){\left(\kappa_{2}-\kappa_{1}\right)}^{\alpha +1}\sum_{n=0}^{\infty }\frac{1}{p^{\alpha +1}}{\left(\frac{q}{p}\right)}^{n\left(\alpha +1\right)}\\ \; & =\frac{{\left(\kappa_{2}-\kappa_{1}\right)}^{\alpha +1}}{{\left[\alpha +1\right]}_{p,q}}. \end{array}$$

Similarly, we can compute the second integral by using the Definition 10, for more details see in [18]. □

The main objective of this paper is to present some new (p,q) estimates of trapezoidal type inequalities for strongly convex and quasi-convex functions and show the relationship between the results given herein. Some examples are given to illustrate the investigated results. Finally, conclusion part is given at the end.

## 2. Trapezoidal Type Inequalities for (*p*,*q*)-Quantum Integrals

We are now providing new trapezoidal type inequalities for functions whose absolute value of first ${\left(p,q\right)}_{\kappa_{1}}$- and ${\left(p,q\right)}^{\kappa_{2}}$-derivatives are strongly convex functions with modulus χ≥1. To prove our main results, we will initially suggest the following useful lemmas.

**Lemma** **4.** *Suppose that $K:\left[\kappa_{1},\kappa_{2}\right]\to R$ is a ${\left(p,q\right)}_{\kappa_{1}}$-differentiable function on $\left(\kappa_{1},\kappa_{2}\right)$. If ${}_{\kappa_{1}}D_{p,q}K$ is a ${\left(p,q\right)}_{\kappa_{1}}$-integrable on $\left(\kappa_{1},\kappa_{2}\right).$ Then, the following identity holds:*

(18) $$\begin{array}{c} \frac{1}{p\left(\kappa_{2}-\kappa_{1}\right)}\int_{\kappa_{1}}^{\left(1-p\right)\kappa_{1}+p\kappa_{2}}K\left(x\right){\;}_{\kappa_{1}}d_{p,q}x-\frac{qK\left(\kappa_{1}\right)+pK\left(\kappa_{2}\right)}{{\left[2\right]}_{p,q}}\\ =\frac{q\left(\kappa_{2}-\kappa_{1}\right)}{2}\int_{0}^{1}\int_{0}^{1}\left(\epsilon -\tau \right)\;\left[{}_{\kappa_{1}}D_{p,q}K\left(\left(1-\tau \right)\kappa_{1}+\tau \kappa_{2}\right)-{\;}_{\kappa_{1}}D_{p,q}K\left(\left(1-\epsilon \right)\kappa_{1}+\epsilon \kappa_{2}\right)\right]\;\;d_{p,q}\tau \;\;d_{p,q}\epsilon . \end{array}$$

**Proof.**  By using Definitions 8 and 10, we have

(19) $$\begin{array}{c} \int_{0}^{1}\int_{0}^{1}\left(\epsilon -\tau \right)\left[{}_{\kappa_{1}}D_{p,q}K\left(\left(1-\tau \right)\kappa_{1}+p\tau \kappa_{2}\right){-}_{\kappa_{1}}D_{p,q}K\left(\left(1-\epsilon \right)\kappa_{1}+\epsilon \kappa_{2}\right)\right]\;\;d_{p,q}\tau \;d_{p,q}\epsilon \\ =\int_{0}^{1}\int_{0}^{1}\left(\epsilon -\tau \right)\;\left[\frac{K\left(\left(1-\tau \right)\kappa_{1}+p\tau \kappa_{2}\right)-K\left(\left(1-q\tau \right)\kappa_{1}+q\tau \kappa_{2}\right)}{\left(p-q\right)\left(\kappa_{2}-\kappa_{1}\right)\tau }\right.\\ \left.-\;\frac{K\left(\left(1-p\epsilon \right)\kappa_{1}+\epsilon \kappa_{2}\right)-K\left(\left(1-q\epsilon \right)\kappa_{1}+q\epsilon \kappa_{2}\right)}{\left(p-q\right)\left(\kappa_{2}-\kappa_{1}\right)\epsilon }\right]\;\;d_{p,q}\tau \;d_{p,q}\epsilon \\ =\int_{0}^{1}\int_{0}^{1}\frac{\epsilon \left[K\left(\left(1-p\tau \right)\kappa_{1}+p\tau \kappa_{2}\right)-K\left(\left(1-q\tau \right)\kappa_{1}+q\tau \kappa_{2}\right)\right]}{\left(1-q\right)\left(\kappa_{2}-\kappa_{1}\right)\tau }\;\;d_{p,q}\tau \;d_{p,q}\epsilon \\ -\int_{0}^{1}\int_{0}^{1}\frac{K\left(\left(1-p\epsilon \right)\kappa_{1}+p\epsilon \kappa_{2}\right)-K\left(\left(1-q\epsilon \right)\kappa_{1}+q\epsilon \kappa_{2}\right)}{\left(p-q\right)\left(\kappa_{2}-\kappa_{1}\right)}\;\;d_{p,q}\tau \;d_{p,q}\epsilon \\ -\int_{0}^{1}\int_{0}^{1}\frac{K\left(\left(1-p\tau \right)\kappa_{1}+p\tau \kappa_{2}\right)-K\left(\left(1-q\tau \right)\kappa_{1}+q\tau \kappa_{2}\right)}{\left(p-q\right)\left(\kappa_{2}-\kappa_{1}\right)}\;\;d_{p,q}\tau \;d_{p,q}\epsilon \\ +\int_{0}^{1}\int_{0}^{1}\;\frac{\tau \left[K\left(\left(1-p\epsilon \right)\kappa_{1}+p\epsilon \kappa_{2}\right)-K\left(\left(1-q\epsilon \right)\kappa_{1}+q\epsilon \kappa_{2}\right)\right]}{\left(1-q\right)\left(\kappa_{2}-\kappa_{1}\right)\epsilon }\;\;d_{p,q}\tau \;d_{p,q}\epsilon . \end{array}$$

We observe that

(20) $$\begin{array}{c} \int_{0}^{1}\int_{0}^{1}\frac{\epsilon \left[K\left(\left(1-p\tau \right)\kappa_{1}+p\tau \kappa_{2}\right)-K\left(\left(1-q\tau \right)\kappa_{1}+q\tau \kappa_{2}\right)\right]}{\left(1-q\right)\left(\kappa_{2}-\kappa_{1}\right)\tau }\;\;d_{p,q}\tau {\;}_{0}d_{q}\epsilon \\ =\int_{0}^{1}\epsilon \;d_{p,q}\epsilon \int_{0}^{1}\frac{K\left(\left(1-p\tau \right)\kappa_{1}+p\tau \kappa_{2}\right)}{\left(p-q\right)\left(\kappa_{2}-\kappa_{1}\right)\tau }{\;}_{0}d_{q}\tau -\int_{0}^{1}\epsilon \;d_{p,q}\epsilon \int_{0}^{1}\frac{K\left(\left(1-q\tau \right)\kappa_{1}+q\tau \kappa_{2}\right)}{\left(p-q\right)\left(\kappa_{2}-\kappa_{1}\right)\tau }\;\;d_{p,q}\tau \\ =\frac{\left(p-q\right)}{\kappa_{2}-\kappa_{1}}\sum_{n=0}^{\infty }\frac{q^{2n}}{p^{2n+2}}\left[\sum_{n=0}^{\infty }K\left(\left(1-\frac{q^{n}}{p^{n}}\right)\kappa_{1}+\frac{q^{n}}{p^{n}}\kappa_{2}\right)-\sum_{n=0}^{\infty }K\left(\left(1-\frac{q^{n+1}}{p^{n+1}}\right)\kappa_{1}+\frac{q^{n+1}}{p^{n+1}}\kappa_{2}\right)\right]\\ =\frac{1}{{\left[2\right]}_{p,q}\left(\kappa_{2}-\kappa_{1}\right)}\left[\sum_{n=0}^{\infty }K\left(\left(1-\frac{q^{n}}{p^{n}}\right)\kappa_{1}+\frac{q^{n}}{p^{n}}\kappa_{2}\right)-\sum_{n=1}^{\infty }K\left(\left(1-\frac{q^{n}}{p^{n}}\right)\kappa_{1}+\frac{q^{n}}{p^{n}}\kappa_{2}\right)\right]\\ =\frac{K\left(\kappa_{2}\right)-K\left(\kappa_{1}\right)}{{\left[2\right]}_{p,q}\left(\kappa_{2}-\kappa_{1}\right)}. \end{array}$$

and

(21) $$\begin{array}{c} \int_{0}^{1}\int_{0}^{1}\frac{K\left(\left(1-p\epsilon \right)\kappa_{1}+p\epsilon \kappa_{2}\right)-K\left(\left(1-qs\right)\kappa_{1}+q\epsilon \kappa_{2}\right)}{\left(p-q\right)\left(\kappa_{2}-\kappa_{1}\right)}\;\;d_{p,q}\tau \;d_{p,q}\epsilon \\ \;=\int_{0}^{1}\;\;d_{p,q}\tau \int_{0}^{1}\frac{K\left(\left(1-p\epsilon \right)\kappa_{1}+p\epsilon \kappa_{2}\right)}{\left(p-q\right)\left(\kappa_{2}-\kappa_{1}\right)}{\;}_{0}d_{q}\epsilon -\int_{0}^{1}\;\;d_{p,q}\tau \int_{0}^{1}\frac{K\left(\left(1-qs\right)\kappa_{1}+q\epsilon \kappa_{2}\right)}{\left(p-q\right)\left(\kappa_{2}-\kappa_{1}\right)}{\;}_{0}d_{q}\epsilon \\ \;=\frac{\left(p-q\right)}{\kappa_{2}-\kappa_{1}}\sum_{n=0}^{\infty }\frac{q^{n}}{p^{n+1}}\left[\sum_{n=0}^{\infty }\frac{q^{n}}{p^{n+1}}K\left(\left(1-q^{n}\right)\kappa_{1}+q^{n}\kappa_{2}\right)-\sum_{n=0}^{\infty }\frac{q^{n}}{p^{n+1}}K\left(\left(1-\frac{q^{n+1}}{p^{n+1}}\right)\kappa_{1}+\frac{q^{n+1}}{p^{n+1}}\kappa_{2}\right)\right]\\ \;=\frac{1}{\kappa_{2}-\kappa_{1}}\left[\frac{1}{p}\sum_{n=0}^{\infty }\frac{q^{n}}{p^{n}}K\left(\left(1-\frac{q^{n}}{p^{n}}\right)\kappa_{1}+\frac{q^{n}}{p^{n}}\kappa_{2}\right)-\frac{1}{q}\sum_{n=1}^{\infty }\frac{q^{n}}{p^{n}}K\left(\left(1-\frac{q^{n}}{p^{n}}\right)\kappa_{1}+\frac{q^{n}}{p^{n}}\kappa_{2}\right)\right]\\ \;=\frac{1}{\kappa_{2}-\kappa_{1}}\left[\frac{1}{q}K\left(\kappa_{2}\right)-\left(\frac{1}{q}-\frac{1}{p}\right)\sum_{n=0}^{\infty }\frac{q^{n}}{p^{n}}K\left(\left(1-\frac{q^{n}}{p^{n}}\right)\kappa_{1}+\frac{q^{n}}{p^{n}}\kappa_{2}\right)\right]\\ =-\frac{1}{pq{\left(\kappa_{2}-\kappa_{1}\right)}^{2}}\int_{\kappa_{1}}^{\left(1-p\right)\kappa_{1}+p\kappa_{2}}K\left(x\right){\;}_{\kappa_{1}}d_{p,q}x+\frac{K\left(\kappa_{2}\right)}{q\left(\kappa_{2}-\kappa_{1}\right)}. \end{array}$$

Similarly,

(22) $$\begin{array}{c} \int_{0}^{1}\int_{0}^{1}\frac{K\left(\left(1-p\tau \right)\kappa_{1}+p\tau \kappa_{2}\right)-K\left(\left(1-q\tau \right)\kappa_{1}+q\tau \kappa_{2}\right)}{\left(p-q\right)\left(\kappa_{2}-\kappa_{1}\right)}\;\;d_{p,q}\tau \;d_{p,q}\epsilon \\ =\int_{0}^{1}\;d_{p,q}\epsilon \int_{0}^{1}\frac{K\left(\left(1-p\tau \right)\kappa_{1}+p\tau \kappa_{2}\right)-K\left(\left(1-q\tau \right)\kappa_{1}+q\tau \kappa_{2}\right)}{\left(p-q\right)\left(\kappa_{2}-\kappa_{1}\right)}\;\;d_{p,q}\tau \\ =-\frac{1}{pq{\left(\kappa_{2}-\kappa_{1}\right)}^{2}}\int_{\kappa_{1}}^{\left(1-p\right)\kappa_{1}+p\kappa_{2}}K\left(x\right){\;}_{\kappa_{1}}d_{p,q}x+\frac{K\left(\kappa_{2}\right)}{q\left(\kappa_{2}-\kappa_{1}\right)}. \end{array}$$

and

(23) $$\begin{array}{c} \int_{0}^{1}\int_{0}^{1}\;\frac{\tau \left[K\left(\left(1-p\epsilon \right)\kappa_{1}+p\epsilon \kappa_{2}\right)-K\left(\left(1-q\epsilon \right)\kappa_{1}+q\epsilon \kappa_{2}\right)\right]}{\left(1-q\right)\left(\kappa_{2}-\kappa_{1}\right)\epsilon }\;\;d_{p,q}\tau \;d_{p,q}\epsilon \\ =\int_{0}^{1}\tau \;\;d_{p,q}\tau \int_{0}^{1}\;\frac{K\left(\left(1-p\epsilon \right)\kappa_{1}+p\epsilon \kappa_{2}\right)-K\left(\left(1-q\epsilon \right)\kappa_{1}+q\epsilon \kappa_{2}\right)}{\left(p-q\right)\left(\kappa_{2}-\kappa_{1}\right)\epsilon }\;d_{p,q}\epsilon \\ =\frac{K\left(\kappa_{2}\right)-K\left(\kappa_{1}\right)}{{\left[2\right]}_{p,q}\left(\kappa_{2}-\kappa_{1}\right)}. \end{array}$$

The equalities (20)–(23) give

(24) $$\begin{array}{c} \int_{0}^{1}\int_{0}^{1}\left(\epsilon -\tau \right)\;\left[{}_{\kappa_{1}}D_{p,q}K\left(\left(1-\tau \right)\kappa_{1}+p\tau \kappa_{2}\right){-}_{\kappa_{1}}D_{p,q}K\left(\left(1-p\epsilon \right)\kappa_{1}+p\epsilon \kappa_{2}\right)\right]\;\;d_{p,q}\tau \;d_{p,q}\epsilon \\ =\frac{2}{pq{\left(\kappa_{2}-\kappa_{1}\right)}^{2}}\int_{\kappa_{1}}^{\left(1-p\right)\kappa_{1}+p\kappa_{2}}K\left(x\right){\;}_{\kappa_{1}}d_{p,q}x-\frac{2K\left(\kappa_{2}\right)}{q\left(\kappa_{2}-\kappa_{1}\right)}+\frac{2\left[K\left(\kappa_{2}\right)-K\left(\kappa_{1}\right)\right]}{{\left[2\right]}_{p,q}\left(\kappa_{2}-\kappa_{1}\right)}. \end{array}$$

Multiplying both sides of (24) by $\frac{q\left(\kappa_{2}-\kappa_{1}\right)}{2}$, we get (18). □

**Lemma** **5.** *Suppose that $K:\left[\kappa_{1},\kappa_{2}\right]\to R$ is a ${\left(p,q\right)}^{\kappa_{2}}$-differentiable function on $\left(\kappa_{1},\kappa_{2}\right)$. If ${}^{\kappa_{2}}D_{p,q}K$ is a ${\left(p,q\right)}^{\kappa_{2}}$-integrable on $\left(\kappa_{1},\kappa_{2}\right)$. Then, the following identity holds:*

(25) $$\begin{array}{c} \frac{1}{p\left(\kappa_{2}-\kappa_{1}\right)}\int_{\left(1-p\right)\kappa_{2}+p\kappa_{1}}^{\kappa_{2}}K\left(x\right){\;}^{\kappa_{2}}d_{p,q}x-\frac{pK\left(\kappa_{1}\right)+qK\left(\kappa_{2}\right)}{{\left[2\right]}_{p,q}}\\ \;=\frac{q\left(\kappa_{2}-\kappa_{1}\right)}{2}\int_{0}^{1}\int_{0}^{1}\left(\epsilon -\tau \right)\;\left[{}^{\kappa_{2}}D_{p,q}K\left(\left(1-\tau \right)\kappa_{2}+\tau \kappa_{1}\right)-{\;}^{\kappa_{2}}D_{p,q}K\left(\left(1-\epsilon \right)\kappa_{2}+\epsilon \kappa_{1}\right)\right]\;\;d_{p,q}\tau \;\;d_{p,q}\epsilon . \end{array}$$

**Proof.**  The proof is directly followed by Definitions 9 and 11. We omit the details. □

**Theorem** **7.** *If we suppose that all of the criteria of Lemma 4 are satisfied, then the resulting inequality, shows that ${\left|{}_{\kappa_{1}}D_{p,q}K\right|}^{\sigma }$ is a strongly convex functions on $\left[\kappa_{1},\kappa_{2}\right]$ with modulus χ≥1 for σ≥1, then*

(26) $$\begin{array}{c} \left|\frac{1}{p\left(\kappa_{2}-\kappa_{1}\right)}\int_{\kappa_{1}}^{\left(1-p\right)\kappa_{1}+p\kappa_{2}}K\left(x\right){\;}_{\kappa_{1}}d_{p,q}x-\frac{qK\left(\kappa_{1}\right)+pK\left(\kappa_{2}\right)}{{\left[2\right]}_{p,q}}\right|\leq q\left(\kappa_{2}-\kappa_{1}\right){\left[W_{5}\left(p,q\right)\right]}^{1-\frac{1}{\sigma }}\\ \;\times {\left[W_{1}\left(p,q\right){\left|{}_{\kappa_{1}}D_{p,q}K\left(\kappa_{1}\right)\right|}^{\sigma }+W_{2}\left(p,q\right){\left|{}_{\kappa_{1}}D_{p,q}K\left(\kappa_{2}\right)\right|}^{\sigma }-\chi {\left(\kappa_{2}-\kappa_{1}\right)}^{2}W_{4}\left(p,q\right)\right]}^{\frac{1}{\sigma }}, \end{array}$$

*where*

$$\begin{array}{cc} \; & W_{1}\left(p,q\right)=\frac{{\left[2\right]}_{p,q}^{2}\left({\left[4\right]}_{p,q}+2\right)-2{\left[2\right]}_{p,q}\left({\left[3\right]}_{p,q}+{\left[4\right]}_{p,q}\right)+{\left[3\right]}_{p,q}{\left[4\right]}_{p,q}}{{\left[2\right]}_{p,q}^{2}{\left[3\right]}_{p,q}{\left[4\right]}_{p,q}}\\ \; & W_{2}\left(p,q\right)=\frac{2{\left[2\right]}_{p,q}\left({\left[3\right]}_{p,q}-{\left[2\right]}_{p,q}\right)+{\left[4\right]}_{p,q}\left({\left[2\right]}_{p,q}^{2}-{\left[3\right]}_{p,q}\right)}{{\left[2\right]}_{p,q}^{2}{\left[3\right]}_{p,q}{\left[4\right]}_{p,q}}\\ \; & W_{3}\left(p,q\right)=\frac{2{\left[2\right]}_{p,q}\left[{\left[4\right]}_{p,q}-{\left[3\right]}_{p,q}\right]+{\left[5\right]}_{p,q}\left[{\left[2\right]}_{p,q}{\left[3\right]}_{p,q}-{\left[4\right]}_{p,q}\right]}{{\left[2\right]}_{p,q}{\left[3\right]}_{p,q}{\left[4\right]}_{p,q}{\left[5\right]}_{p,q}}\\ \; & W_{4}\left(p,q\right)=\frac{2{\left[2\right]}_{p,q}\left({\left[3\right]}_{p,q}-{\left[2\right]}_{p,q}\right)+{\left[4\right]}_{p,q}\left({\left[2\right]}_{p,q}^{2}-{\left[3\right]}_{p,q}\right)}{{\left[2\right]}_{p,q}^{2}{\left[3\right]}_{p,q}{\left[4\right]}_{p,q}}\\ \; & -\frac{2{\left[2\right]}_{p,q}\left[{\left[4\right]}_{p,q}-{\left[3\right]}_{p,q}\right]+{\left[5\right]}_{p,q}\left[{\left[2\right]}_{p,q}{\left[3\right]}_{p,q}-{\left[4\right]}_{p,q}\right]}{{\left[2\right]}_{p,q}{\left[3\right]}_{p,q}{\left[4\right]}_{p,q}{\left[5\right]}_{p,q}}\\ \; & W_{5}\left(p,q\right)=\frac{2\left({\left[2\right]}_{p,q}-1\right)}{{\left[2\right]}_{p,q}{\left[3\right]}_{p,q}}. \end{array}$$

**Proof.**  Taking modulus on Equation (18) and using the power-mean inequality, we have

(27) $$\begin{array}{c} \left|\frac{1}{p\left(\kappa_{2}-\kappa_{1}\right)}\int_{\kappa_{1}}^{\left(1-p\right)\kappa_{1}+p\kappa_{2}}K\left(x\right){\;}_{\kappa_{1}}d_{p,q}x-\frac{qK\left(\kappa_{1}\right)+pK\left(\kappa_{2}\right)}{{\left[2\right]}_{p,q}}\right|\\ \leq \frac{q\left(\kappa_{2}-\kappa_{1}\right)}{2}{\left(\int_{0}^{1}\int_{0}^{1}\left|\epsilon -\tau \right|\;d_{p,q}\tau \;\;d_{p,q}\epsilon \right)}^{1-\frac{1}{\sigma }}\;\\ \times \left\{{\left(\int_{0}^{1}\int_{0}^{1}\left|\epsilon -\tau \right|{\left|{\;}_{\kappa_{1}}D_{p,q}K\left(\left(1-\tau \right)\kappa_{1}+\tau \kappa_{2}\right)\right|}^{\sigma }\;\;d_{p,q}\tau \;\;d_{p,q}\epsilon \right)}^{\frac{1}{\sigma }}\right.\\ \left.+{\left(\int_{0}^{1}\int_{0}^{1}\left|\epsilon -\tau \right|{\left|{\;}_{\kappa_{1}}D_{p,q}K\left(\left(1-\epsilon \right)\kappa_{1}+\epsilon \kappa_{2}\right)\right|}^{\sigma }\;\;d_{p,q}\tau \;\;d_{p,q}\epsilon \right)}^{\frac{1}{\sigma }}\right\}. \end{array}$$

Using the strongly convexity of ${\left|{}_{\kappa_{1}}D_{p,q}K\right|}^{\sigma }$ on $\left[\kappa_{1},\kappa_{2}\right]$, we obtain

(28) $$\begin{array}{c} \int_{0}^{1}\int_{0}^{1}\left|\epsilon -\tau \right|{\left|{\;}_{\kappa_{1}}D_{p,q}K\left(\left(1-\tau \right)\kappa_{1}+\tau \kappa_{2}\right)\right|}^{\sigma }\;\;d_{p,q}\tau \;\;d_{p,q}\epsilon \\ \leq {\left|{}_{\kappa_{1}}D_{p,q}K\left(\kappa_{1}\right)\right|}^{\sigma }\int_{0}^{1}\int_{0}^{1}\left|\epsilon -\tau \right|\left(1-\tau \right)\;\;d_{p,q}\tau \;\;d_{p,q}\epsilon \\ +{\left|{}_{\kappa_{1}}D_{p,q}K\left(\kappa_{2}\right)\right|}^{\sigma }\int_{0}^{1}\int_{0}^{1}\left|\epsilon -\tau \right|\tau \;\;d_{p,q}\tau \;\;d_{p,q}\epsilon -\chi {\left(\kappa_{2}-\kappa_{1}\right)}^{2}\int_{0}^{1}\int_{0}^{1}\left|\epsilon -\tau \right|\tau \left(1-\tau \right)\;d_{p,q}\tau \;\;d_{p,q}\epsilon . \end{array}$$

By using Definition 10, we get $2\left(\frac{1}{y}-\frac{1}{z}\right)\frac{1}{w}-\left(\frac{1}{xy}-\frac{1}{z}\right)=-\frac{1}{wxyz}\left(wz+2xy-2xz-wxy\right)$

(29) $$\begin{array}{c} W_{1}\left(p,q\right)=\int_{0}^{1}\int_{0}^{1}\left|\epsilon -\tau \right|\left(1-\tau \right)\;\;d_{p,q}\tau \;\;d_{p,q}\epsilon \\ =\int_{0}^{1}\left(2\int_{0}^{\epsilon }\left(\epsilon -\tau \right)\left(1-\tau \right)\;\;d_{p,q}\tau -\int_{0}^{1}\left(\epsilon -\tau \right)\left(1-\tau \right)\;\;d_{p,q}\tau \right)\;d_{p,q}\epsilon \\ =\int_{0}^{1}\left(2\int_{0}^{\epsilon }\left(\epsilon -\epsilon \tau -\tau +\tau^{2}\right)\;d_{p,q}\tau -\int_{0}^{1}\left(\epsilon -\epsilon \tau -\tau +\tau^{2}\right)d_{p,q}\tau \right)\;d_{p,q}\epsilon \\ =\int_{0}^{1}\left(2\left(\epsilon^{2}-\frac{\epsilon^{3}}{{\left[2\right]}_{p,q}}-\frac{\epsilon^{2}}{{\left[2\right]}_{p,q}}+\frac{\epsilon^{3}}{{\left[3\right]}_{p,q}}\right)-\left(\epsilon -\frac{\epsilon }{{\left[2\right]}_{p,q}}-\frac{1}{{\left[2\right]}_{p,q}}+\frac{1}{{\left[3\right]}_{p,q}}\right)\right)\;d_{p,q}\epsilon \\ =2\left(\frac{1}{{\left[3\right]}_{p,q}}-\frac{1}{{\left[2\right]}_{p,q}{\left[4\right]}_{p,q}}-\frac{1}{{\left[2\right]}_{p,q}{\left[3\right]}_{p,q}}+\frac{1}{{\left[3\right]}_{p,q}{\left[4\right]}_{p,q}}\right)\\ -\left(\frac{1}{{\left[2\right]}_{p,q}}-\frac{1}{{\left[2\right]}_{p,q}^{2}}-\frac{1}{{\left[2\right]}_{p,q}}+\frac{1}{{\left[3\right]}_{p,q}}\right)\\ =\frac{{\left[2\right]}_{p,q}^{2}\left({\left[4\right]}_{p,q}+2\right)-2{\left[2\right]}_{p,q}\left({\left[3\right]}_{p,q}+{\left[4\right]}_{p,q}\right)+{\left[3\right]}_{p,q}{\left[4\right]}_{p,q}}{{\left[2\right]}_{p,q}^{2}{\left[3\right]}_{p,q}{\left[4\right]}_{p,q}}. \end{array}$$

(30) $$\begin{array}{c} W_{2}\left(p,q\right)=\int_{0}^{1}\int_{0}^{1}\left|\epsilon -\tau \right|\tau \;\;d_{p,q}\tau \;\;d_{p,q}\epsilon \\ =\int_{0}^{1}\left[-2\epsilon^{3}\frac{\left[{\left[2\right]}_{p,q}-{\left[3\right]}_{p,q}\right]}{{\left[2\right]}_{p,q}{\left[3\right]}_{p,q}}-\frac{\epsilon }{{\left[2\right]}_{p,q}}+\frac{1}{{\left[3\right]}_{p,q}}\right]\;d_{p,q}\epsilon \\ =\frac{2{\left[2\right]}_{p,q}\left[{\left[3\right]}_{p,q}-{\left[2\right]}_{p,q}\right]+{\left[4\right]}_{p,q}\left[{\left[2\right]}_{p,q}^{2}-{\left[3\right]}_{p,q}\right]}{{\left[2\right]}_{p,q}^{2}{\left[3\right]}_{p,q}{\left[4\right]}_{p,q}}. \end{array}$$

(31) $$\begin{array}{c} W_{3}\left(p,q\right)=\int_{0}^{1}\int_{0}^{1}\left|\epsilon -\tau \right|\tau^{2}\;\;d_{p,q}\tau \;\;d_{p,q}\epsilon \\ =\int_{0}^{1}\left(2\int_{0}^{\epsilon }\left(\epsilon -\tau \right)\tau^{2}\;\;d_{p,q}\tau -\int_{0}^{1}\left(\epsilon -\tau \right)\tau^{2}\;\;d_{p,q}\tau \right)d_{p,q}\epsilon \\ =\int_{0}^{1}\left(2\int_{0}^{\epsilon }\left(\epsilon \tau^{2}-\tau^{3}\right)\;\;d_{p,q}\tau -\int_{0}^{1}\left(\epsilon \tau^{2}-\tau^{3}\right)d_{p,q}\tau \right)d_{p,q}\epsilon \\ =\int_{0}^{1}\left[2\left(\frac{1}{{\left[3\right]}_{p,q}}-\frac{1}{{\left[4\right]}_{p,q}}\right)\epsilon^{4}-\left(\frac{\epsilon }{{\left[3\right]}_{p,q}}-\frac{1}{{\left[4\right]}_{p,q}}\right)\right]\;d_{p,q}\epsilon \\ =2\left(\frac{1}{{\left[3\right]}_{p,q}}-\frac{1}{{\left[4\right]}_{p,q}}\right)\frac{1}{{\left[5\right]}_{p,q}}-\left(\frac{1}{{\left[2\right]}_{p,q}{\left[3\right]}_{p,q}}-\frac{1}{{\left[4\right]}_{p,q}}\right)\\ =\frac{2{\left[2\right]}_{p,q}\left[{\left[4\right]}_{p,q}-{\left[3\right]}_{p,q}\right]+{\left[5\right]}_{p,q}\left[{\left[2\right]}_{p,q}{\left[3\right]}_{p,q}-{\left[4\right]}_{p,q}\right]}{{\left[2\right]}_{p,q}{\left[3\right]}_{p,q}{\left[4\right]}_{p,q}{\left[5\right]}_{p,q}}. \end{array}$$

(32) $$\begin{array}{cc} W_{4}\left(p,q\right) & =\int_{0}^{1}\int_{0}^{1}\left|\epsilon -\tau \right|\tau \left(1-\tau \right)\;\;d_{p,q}\tau \;\;d_{p,q}\epsilon \end{array}$$

(33) $$\begin{array}{cc} \; & =W_{2}\left(p,q\right)-W_{3}\left(p,q\right) \end{array}$$

(34) $$\begin{array}{cc} \; & =\frac{2{\left[2\right]}_{p,q}\left({\left[3\right]}_{p,q}-{\left[2\right]}_{p,q}\right)+{\left[4\right]}_{p,q}\left({\left[2\right]}_{p,q}^{2}-{\left[3\right]}_{p,q}\right)}{{\left[2\right]}_{p,q}^{2}{\left[3\right]}_{p,q}{\left[4\right]}_{p,q}} \end{array}$$

(35) $$\begin{array}{cc} \; & -\frac{2{\left[2\right]}_{p,q}\left[{\left[4\right]}_{p,q}-{\left[3\right]}_{p,q}\right]+{\left[5\right]}_{p,q}\left[{\left[2\right]}_{p,q}{\left[3\right]}_{p,q}-{\left[4\right]}_{p,q}\right]}{{\left[2\right]}_{p,q}{\left[3\right]}_{p,q}{\left[4\right]}_{p,q}{\left[5\right]}_{p,q}}. \end{array}$$

Applying (29)–(32) in (28), we get

(36) $$\begin{array}{c} \int_{0}^{1}\int_{0}^{1}\left|\epsilon -\tau \right|{\left|{}_{\kappa_{1}}D_{p,q}K\left(\left(1-\epsilon \right)\kappa_{1}+\epsilon \kappa_{2}\right)\right|}^{\sigma }\;\;d_{p,q}\tau \;\;d_{p,q}\epsilon \\ \leq {\left|{}_{\kappa_{1}}D_{p,q}K\left(\kappa_{1}\right)\right|}^{\sigma }W_{1}\left(p,q\right)\\ +{\left|{}_{\kappa_{1}}D_{p,q}K\left(\kappa_{2}\right)\right|}^{\sigma }W_{2}\left(p,q\right)-\chi {\left(\kappa_{2}-\kappa_{1}\right)}^{2}W_{4}\left(p,q\right). \end{array}$$

Similarly, we also observe that

(37) $$\begin{array}{c} \int_{0}^{1}\int_{0}^{1}\left|\epsilon -\tau \right|{\left|{}_{\kappa_{1}}D_{p,q}K\left(\left(1-\epsilon \right)\kappa_{1}+\epsilon \kappa_{2}\right)\right|}^{\sigma }\;\;d_{p,q}\tau \;\;d_{p,q}\epsilon \\ \leq {\left|{}_{\kappa_{1}}D_{p,q}K\left(\kappa_{1}\right)\right|}^{\sigma }W_{1}\left(p,q\right)\\ +{\left|{}_{\kappa_{1}}D_{p,q}K\left(\kappa_{2}\right)\right|}^{\sigma }W_{2}\left(p,q\right)-\chi {\left(\kappa_{2}-\kappa_{1}\right)}^{2}W_{4}\left(p,q\right). \end{array}$$

We also have

(38) $$\begin{array}{c} W_{5}\left(p,q\right)=\int_{0}^{1}\int_{0}^{1}\left|\epsilon -\tau \right|\;d_{p,q}\tau \;\;d_{p,q}\epsilon =\int_{0}^{1}\left(-2\int_{0}^{\epsilon }\left(\tau -\epsilon \right)\;d_{p,q}\tau +\int_{0}^{1}\left(\tau -\epsilon \right)\;d_{p,q}\tau \right)\;\;d_{p,q}\epsilon \\ =\int_{0}^{1}\left(-2\epsilon^{2}\frac{\left[1-{\left[2\right]}_{p,q}\right]}{{\left[2\right]}_{p,q}}-\epsilon +\frac{1}{{\left[2\right]}_{p,q}}\right)\;\;d_{p,q}\epsilon =\frac{2\left[{\left[2\right]}_{p,q}-1\right]}{{\left[2\right]}_{p,q}{\left[3\right]}_{p,q}}. \end{array}$$

Applying (36)–(38) in (27), we obtain the desired inequality. □

**Corollary** **2.** *If σ=1 together with the assumptions of Theorem 7, we obtain*

(39) $$\begin{array}{c} \left|\frac{1}{\kappa_{2}-\kappa_{1}}\int_{\kappa_{1}}^{\left(1-p\right)\kappa_{1}+p\kappa_{2}}K\left(x\right){\;}_{\kappa_{1}}d_{p,q}x-\frac{qK\left(\kappa_{1}\right)+pK\left(\kappa_{2}\right)}{{\left[2\right]}_{p,q}}\right|\leq q\left(\kappa_{2}-\kappa_{1}\right)\\ \times \left[W_{1}\left(p,q\right)\left|{}_{\kappa_{1}}D_{p,q}K\left(\kappa_{1}\right)\right|+W_{2}\left(p,q\right)\left|{}_{\kappa_{1}}D_{p,q}K\left(\kappa_{2}\right)\right|-\chi {\left(\kappa_{2}-\kappa_{1}\right)}^{2}W_{4}\left(p,q\right)\right], \end{array}$$

*where $W_{1}\left(p,q\right),W_{2}\left(p,q\right)$ and $W_{4}\left(p,q\right)$ are defined in Theorem 7.*

**Corollary** **3.** *As p=1 and $q\to 1^{-}$ in Theorem 7, we get the inequality*

(40) $$\begin{array}{c} \left|\frac{1}{\kappa_{2}-\kappa_{1}}\int_{\kappa_{1}}^{\kappa_{2}}K\left(x\right)dx-\frac{K\left(\kappa_{1}\right)+K\left(\kappa_{2}\right)}{2}\right|\leq \left(\kappa_{2}-\kappa_{1}\right){\left(\frac{1}{3}\right)}^{1-\frac{1}{\sigma }}{\left[\frac{{\left|K^{\prime }\left(\kappa_{1}\right)\right|}^{\sigma }+{\left|K^{\prime }\left(\kappa_{2}\right)\right|}^{\sigma }}{6}-\frac{\chi {\left(\kappa_{2}-\kappa_{1}\right)}^{2}}{20}\right]}^{\frac{1}{\sigma }}. \end{array}$$

**Corollary** **4.** *Suppose that the assumptions of Theorem 7 with σ=1,p=1 and letting $q\to 1^{-}$, we obtain the inequality*

(41) $$\left|\frac{1}{\kappa_{2}-\kappa_{1}}\int_{\kappa_{1}}^{\kappa_{2}}K\left(x\right)dx-\frac{K\left(\kappa_{1}\right)+K\left(\kappa_{2}\right)}{2}\right|\leq \left(\kappa_{2}-\kappa_{1}\right)\left[\frac{\left|K^{\prime }\left(\kappa_{1}\right)\right|+\left|K^{\prime }\left(\kappa_{2}\right)\right|}{6}-\frac{\chi {\left(\kappa_{2}-\kappa_{1}\right)}^{2}}{20}\right].$$

**Theorem** **8.** *If we suppose that all of the criteria of Lemma 4 are satisfied, then the resulting inequality, shows that ${\left|{}_{\kappa_{1}}D_{p,q}K\right|}^{\sigma_{2}}$ is a strongly convex functions on $\left[\kappa_{1},\kappa_{2}\right]$ with modulus χ≥1 for $\frac{1}{\sigma_{1}}+\frac{1}{\sigma_{2}}=1,$ then*

$$\left|\frac{1}{p\left(\kappa_{2}-\kappa_{1}\right)}\int_{\kappa_{1}}^{\left(1-p\right)\kappa_{1}+p\kappa_{2}}K\left(x\right){\;}_{\kappa_{1}}d_{p,q}x-\frac{qK\left(\kappa_{1}\right)+pK\left(\kappa_{2}\right)}{{\left[2\right]}_{p,q}}\right|\leq q\left(\kappa_{2}-\kappa_{1}\right){\left[M\left(p,q\right)\right]}^{1-\frac{1}{\sigma_{1}}}$$

(42) $$\times {\left(\frac{\left[{\left[2\right]}_{p,q}-1\right]{\left|{}_{\kappa_{1}}D_{p,q}K\left(\kappa_{1}\right)\right|}^{\sigma_{2}}+{\left|{}_{\kappa_{1}}D_{p,q}K\left(\kappa_{2}\right)\right|}^{\sigma_{2}}}{{\left[2\right]}_{p,q}}-\frac{\chi {\left(\kappa_{2}-\kappa_{1}\right)}^{2}\left[{\left[3\right]}_{p,q}-{\left[2\right]}_{p,q}\right]}{{\left[2\right]}_{p,q}{\left[3\right]}_{p,q}}\right)}^{\frac{1}{\sigma_{2}}},$$

*where*

$$M\left(p,q\right)=\frac{{\left(q-p\right)}^{2}}{\left(q^{\sigma_{1}+1}-p^{\sigma_{1}+1}\right)}\sum_{m=0}^{\infty }{\left(-1\right)}^{m-1}\frac{\left(3+q^{\sigma_{1}-m+1}-q^{m+1}-2q^{p+1}-q^{p+2}\right)\sigma_{1}\left(\sigma_{1}-1\right)\cdots \left(\sigma_{1}-m+1\right)}{m!\left({\left[2\right]}_{p,q}^{\sigma_{1}-m+1}\right)\left(q^{m+1}-p^{m+1}\right)}.$$

**Proof.**  Taking modulus on Equation (18) and using Hölder inequality, we have

(43) $$\begin{array}{c} \left|\frac{1}{\kappa_{2}-\kappa_{1}}\int_{\kappa_{1}}^{\left(1-p\right)\kappa_{1}+p\kappa_{2}}K\left(x\right){\;}_{\kappa_{1}}d_{p,q}x-\frac{qK\left(\kappa_{1}\right)+pK\left(\kappa_{2}\right)}{2}\right|\\ \leq \frac{q\left(\kappa_{2}-\kappa_{1}\right)}{2}{\left(\int_{0}^{1}\int_{0}^{1}{\left|\epsilon -\tau \right|}^{\sigma_{1}}\;\;d_{p,q}\tau \;\;d_{p,q}\epsilon \right)}^{1-\frac{1}{\sigma_{1}}}\;\\ \times \left\{{\left(\int_{0}^{1}\int_{0}^{1}{\left|{}_{\kappa_{1}}D_{p,q}K\left(\left(1-\tau \right)\kappa_{1}+\tau \kappa_{2}\right)\right|}^{\sigma_{2}}\;\;d_{p,q}\tau \;\;d_{p,q}\epsilon \right)}^{\frac{1}{\sigma_{2}}}\right.\\ \left.+{\left(\int_{0}^{1}\int_{0}^{1}{\left|{}_{\kappa_{1}}D_{p,q}K\left(\left(1-\epsilon \right)\kappa_{1}+\epsilon \kappa_{2}\right)\right|}^{\sigma_{2}}\;\;d_{p,q}\tau \;\;d_{p,q}\epsilon \right)}^{\frac{1}{\sigma_{2}}}\right\}. \end{array}$$

We now evaluate the integrals involved in (43). We observe that

(44) $$\begin{array}{c} \int_{0}^{1}\int_{0}^{1}{\left|\epsilon -\tau \right|}^{\sigma_{1}}\;\;d_{p,q}\tau \;\;d_{p,q}\epsilon =\int_{0}^{1}\left(\int_{0}^{\epsilon }{\left(\epsilon -\tau \right)}^{\sigma_{1}}\;\;d_{p,q}\tau \right)\;\;d_{p,q}\epsilon \\ +\int_{0}^{1}\left(\int_{\epsilon }^{1}{\left(\tau -\epsilon \right)}^{\sigma_{1}}\;\;d_{p,q}\tau \right)\;\;d_{p,q}\epsilon =\int_{0}^{1}\left(\int_{0}^{\epsilon }{\left(\epsilon -\tau \right)}^{\sigma_{1}}\;\;d_{p,q}\tau \right)\;\;d_{p,q}\epsilon \\ +\int_{0}^{1}\left(\int_{0}^{\epsilon }{\left(\tau -\epsilon \right)}^{\sigma_{1}}\;\;d_{p,q}\tau \right)\;\;d_{p,q}\epsilon +\int_{0}^{1}\left(\int_{0}^{1}{\left(\tau -\epsilon \right)}^{\sigma_{1}}\;\;d_{p,q}\tau \right)\;\;d_{p,q}\epsilon . \end{array}$$

Consider

(45) $$\begin{array}{c} \int_{0}^{1}\left(\int_{0}^{\epsilon }{\left(\epsilon -\tau \right)}^{\sigma_{1}}\;\;d_{p,q}\tau \right)\;\;d_{p,q}\epsilon \\ =\frac{p-q}{p^{\sigma_{1}+1}-q^{\sigma_{1}+1}}\left[1-\sigma_{1}\frac{1}{{\left[2\right]}_{p,q}}+\frac{\sigma_{1}\left(\sigma_{1}-1\right)}{2!}\frac{1}{{\left[3\right]}_{p,q}}-\cdots \right]\\ =\frac{{\left(p-q\right)}^{2}}{q^{\sigma_{1}+1}-q^{\sigma_{1}+1}}\sum_{m=0}^{\infty }{\left(-1\right)}^{m-1}\frac{\sigma_{1}\left(\sigma_{1}-1\right)\cdots \left(\sigma_{1}-m+1\right)}{m!\left(p^{m+1}-q^{m+1}\right)}, \end{array}$$

(46) $$\begin{array}{c} \int_{0}^{1}\left(\int_{0}^{\epsilon }{\left(\tau -\epsilon \right)}^{\sigma_{1}}\;\;d_{p,q}\tau \right)\;d_{p,q}\epsilon =\int_{0}^{1}\int_{q\tau }^{1}{\left(\tau -\epsilon \right)}^{\sigma_{1}}\;\;d_{p,q}\epsilon \;\;d_{p,q}\tau \\ =\int_{0}^{1}\int_{0}^{1}{\left(\tau -\epsilon \right)}^{\sigma_{1}}\;\;d_{p,q}\epsilon \;d_{p,q}\tau -\int_{0}^{1}\int_{0}^{q\tau }{\left(\tau -\epsilon \right)}^{\sigma_{1}}\;\;d_{p,q}\epsilon \;\;d_{p,q}\tau \\ ={\left(p-q\right)}^{2}\sum_{m=0}^{\infty }{\left(-1\right)}^{m-1}\frac{\sigma_{1}\left(\sigma_{1}-1\right)\cdots \left(\sigma_{1}-m+1\right)}{m!\left({\left[2\right]}_{p,q}^{p-m+1}\right)\left(p^{m+1}-q^{m+1}\right)}\\ -\frac{q{\left(p-q\right)}^{2}}{p^{\sigma_{1}+1}-q^{\sigma_{1}+1}}\sum_{m=0}^{\infty }{\left(-1\right)}^{m-1}\frac{q^{m}\sigma_{1}\left(\sigma_{1}-1\right)\cdots \left(\sigma_{1}-m+1\right)}{m!\left(p^{m+1}-q^{m+1}\right)} \end{array}$$

and

(47) $$\begin{array}{c} \int_{0}^{1}\left(\int_{0}^{1}{\left(\tau -\epsilon \right)}^{\sigma_{1}}\;\;d_{p,q}\tau \right)\;\;d_{p,q}\epsilon =\int_{0}^{1}\left(\int_{0}^{1}{\left(\tau -\epsilon \right)}^{\sigma_{1}}\;\;d_{p,q}\epsilon \right)\;\;d_{p,q}\tau \\ ={\left(p-q\right)}^{2}\sum_{m=0}^{\infty }{\left(-1\right)}^{m-1}\frac{\sigma_{1}\left(\sigma_{1}-1\right)\cdots \left(\sigma_{1}-m+1\right)}{m!\left({\left[2\right]}_{p,q}^{\sigma_{1}-m+1}\right)\left(p^{m+1}-q^{m+1}\right)}. \end{array}$$

Using the strongly convexity of ${\left|{}_{\kappa_{1}}D_{p,q}K\right|}^{\sigma_{2}}$ on $\left[\kappa_{1},\kappa_{2}\right]$, we obtain

(48) $$\begin{array}{c} \int_{0}^{1}\int_{0}^{1}{\left|{}_{\kappa_{1}}D_{p,q}K\left(\left(1-\tau \right)\kappa_{1}+\tau \kappa_{2}\right)\right|}^{\sigma_{2}}\;\;d_{p,q}\tau \;\;d_{p,q}\epsilon \\ \leq {\left|{}_{\kappa_{1}}D_{p,q}K\left(\kappa_{1}\right)\right|}^{\sigma_{2}}\int_{0}^{1}\left(1-\tau \right)\;\;d_{p,q}\tau +{\left|{}_{\kappa_{1}}D_{p,q}K\left(\kappa_{2}\right)\right|}^{\sigma_{2}}\int_{0}^{1}\tau \;\;d_{p,q}\tau \\ -\chi {\left(\kappa_{2}-\kappa_{1}\right)}^{2}\int_{0}^{1}\int_{0}^{1}\left(1-\tau \right)\tau \;\;d_{p,q}\tau \;\;d_{p,q}\epsilon \\ =\frac{\left[{\left[2\right]}_{p,q}-1\right]{\left|{}_{\kappa_{1}}D_{p,q}K\left(\kappa_{1}\right)\right|}^{\sigma_{2}}+{\left|{}_{\kappa_{1}}D_{p,q}K\left(\kappa_{2}\right)\right|}^{\sigma_{2}}}{{\left[2\right]}_{p,q}}-\frac{\chi {\left(\kappa_{2}-\kappa_{1}\right)}^{2}\left[{\left[3\right]}_{p,q}-{\left[2\right]}_{p,q}\right]}{{\left[2\right]}_{p,q}{\left[3\right]}_{p,q}}. \end{array}$$

and similarly, we get

(49) $$\begin{array}{c} \int_{0}^{1}\int_{0}^{1}{\left|{}_{\kappa_{1}}D_{p,q}K\left(\left(1-\epsilon \right)\kappa_{1}+\epsilon \kappa_{2}\right)\right|}^{\sigma_{2}}\;\;d_{p,q}\tau \;\;d_{p,q}\epsilon \\ \leq \frac{\left[{\left[2\right]}_{p,q}-1\right]{\left|{}_{\kappa_{1}}D_{p,q}K\left(\kappa_{1}\right)\right|}^{\sigma_{2}}+{\left|{}_{\kappa_{1}}D_{p,q}K\left(\kappa_{2}\right)\right|}^{\sigma_{2}}}{{\left[2\right]}_{p,q}}-\frac{\chi {\left(\kappa_{2}-\kappa_{1}\right)}^{2}\left[{\left[3\right]}_{p,q}-{\left[2\right]}_{p,q}\right]}{{\left[2\right]}_{p,q}{\left[3\right]}_{p,q}}. \end{array}$$

Making use of (44) and (49) in (43), we get the required result. □

**Theorem** **9.** *If we suppose that all of the criteria of Lemma 4 are satisfied, then the resulting inequality shows that ${\left|{}_{\kappa_{1}}D_{p,q}K\right|}^{\sigma }$ is a strongly quasi-convex functions on $\left[\kappa_{1},\kappa_{2}\right]$ with modulus χ≥1 for σ≥1, then*

(50) $$\begin{array}{c} \left|\frac{1}{p\left(\kappa_{2}-\kappa_{1}\right)}\int_{\kappa_{1}}^{\left(1-p\right)\kappa_{1}+p\kappa_{2}}K\left(x\right){\;}_{\kappa_{1}}d_{p,q}x-\frac{qK\left(\kappa_{1}\right)+pK\left(\kappa_{2}\right)}{{\left[2\right]}_{p,q}}\right|\\ \leq q\left(\kappa_{2}-\kappa_{1}\right){\left[W_{5}\left(p,q\right)\right]}^{1-\frac{1}{\sigma }}{\left[Z\left(p,q\right)W_{5}\left(p,q\right)-\chi {\left(\kappa_{2}-\kappa_{1}\right)}^{2}W_{4}\left(p,q\right)\right]}^{\frac{1}{\sigma }}, \end{array}$$

*where*

$$Z\left(p,q\right)=\max\left\{{\left|{}_{\kappa_{1}}D_{p,q}K\left(\kappa_{1}\right)\right|}^{\sigma },{\left|{}_{\kappa_{1}}D_{p,q}K\left(\kappa_{2}\right)\right|}^{\sigma }\right\},$$

*and $W_{4}\left(p,q\right)$, $W_{5}\left(p,q\right)$ are defined in Theorem 7.*

**Proof.**  Taking modulus on Equation (18) and using the power-mean inequality, we have

(51) $$\begin{array}{c} \left|\frac{1}{p\left(\kappa_{2}-\kappa_{1}\right)}\int_{\kappa_{1}}^{\left(1-p\right)\kappa_{1}+p\kappa_{2}}K\left(x\right){\;}_{\kappa_{1}}d_{p,q}x-\frac{qK\left(\kappa_{1}\right)+pK\left(\kappa_{2}\right)}{{\left[2\right]}_{p,q}}\right|\\ \leq \frac{q\left(\kappa_{2}-\kappa_{1}\right)}{2}{\left(\int_{0}^{1}\int_{0}^{1}\left|\epsilon -\tau \right|\;d_{p,q}\tau \;\;d_{p,q}\epsilon \right)}^{1-\frac{1}{\sigma }}\;\\ \times \left\{{\left(\int_{0}^{1}\int_{0}^{1}\left|\epsilon -\tau \right|{\left|{}_{\kappa_{1}}D_{p,q}K\left(\left(1-\tau \right)\kappa_{1}+\tau \kappa_{2}\right)\right|}^{\sigma }\;\;d_{p,q}\tau \;\;d_{p,q}\epsilon \right)}^{\frac{1}{\sigma }}\right.\\ \left.+{\left(\int_{0}^{1}\int_{0}^{1}\left|\epsilon -\tau \right|{\left|{}_{\kappa_{1}}D_{p,q}K\left(\left(1-\epsilon \right)\kappa_{1}+\epsilon \kappa_{2}\right)\right|}^{\sigma }\;\;d_{p,q}\tau \;\;d_{p,q}\epsilon \right)}^{\frac{1}{\sigma }}\right\}. \end{array}$$

Using the strongly convexity of ${\left|{}_{\kappa_{1}}D_{p,q}K\right|}^{\sigma }$ on $\left[\kappa_{1},\kappa_{2}\right]$, we obtain

(52) $${\left|{}_{\kappa_{1}}D_{p,q}K\left(\left(1-\tau \right)\kappa_{1}+\tau \kappa_{2}\right)\right|}^{\sigma }\;\leq \max\left\{{\left|{}_{\kappa_{1}}D_{p,q}K\left(\kappa_{1}\right)\right|}^{\sigma },{\left|{}_{\kappa_{1}}D_{p,q}K\left(\kappa_{2}\right)\right|}^{\sigma }\right\}-\chi {\left(\kappa_{2}-\kappa_{1}\right)}^{2}\tau \left(1-\tau \right)$$

and

(53) $${\left|{}_{\kappa_{1}}D_{p,q}K\left(\left(1-\epsilon \right)\kappa_{1}+\epsilon \kappa_{2}\right)\right|}^{\sigma }\leq \max\left\{{\left|{}_{\kappa_{1}}D_{p,q}K\left(\kappa_{1}\right)\right|}^{\sigma },{\left|{}_{\kappa_{1}}D_{p,q}K\left(\kappa_{2}\right)\right|}^{\sigma }\right\}-\chi {\left(\kappa_{2}-\kappa_{1}\right)}^{2}\epsilon \left(1-\epsilon \right).$$

Applying (32), (38), (52), and (53) in (51), we get the desired result. □

**Corollary** **5.** *Letting p=1 in Theorem 9, we obtain*

(54) $$\begin{array}{c} \left|\frac{1}{\kappa_{2}-\kappa_{1}}\int_{\kappa_{1}}^{\kappa_{2}}K\left(x\right){\;}_{\kappa_{1}}d_{q}x-\frac{qK\left(\kappa_{1}\right)+K\left(\kappa_{2}\right)}{{\left[2\right]}_{q}}\right|\\ \leq q\left(\kappa_{2}-\kappa_{1}\right){\left(W_{5}\left(1,q\right)\right)}^{1-\frac{1}{\sigma }}{\left(Z\left(1,q\right)\left(W_{5}\left(1,q\right)\right)-\chi {\left(\kappa_{2}-\kappa_{1}\right)}^{2}W_{4}\left(1,q\right)\right)}^{\frac{1}{\sigma }}, \end{array}$$

*where*

$$\begin{array}{cc} \; & W_{4}\left(1,q\right)=\frac{q^{2}\left(q^{4}+q^{3}+q^{2}-q+1\right)}{q^{9}+3q^{8}+6q^{7}+9q^{6}+11q^{5}+11q^{4}+9q^{3}+6q^{2}+3q+1}\\ & W_{5}\left(1,q\right)=\frac{2q}{q^{3}+2q^{2}+2q+1}\\ & Z\left(1,q\right)=\max\left\{{\left|{}_{\kappa_{1}}D_{q}K\left(\kappa_{1}\right)\right|}^{\sigma },{\left|{}_{\kappa_{1}}D_{q}K\left(\kappa_{2}\right)\right|}^{\sigma }\right\}. \end{array}$$

**Corollary** **6.** *Letting p=1 in Theorem 9 together with σ=1, we obtain*

$$\left|\frac{1}{\kappa_{2}-\kappa_{1}}\int_{\kappa_{1}}^{\kappa_{2}}K\left(x\right){\;}_{\kappa_{1}}d_{q}x-\frac{qK\left(\kappa_{1}\right)+K\left(\kappa_{2}\right)}{{\left[2\right]}_{q}}\right|\leq q\left(\kappa_{2}-\kappa_{1}\right)\left(Z\left(1,q\right)\left(W_{5}\left(1,q\right)\right)-\chi {\left(\kappa_{2}-\kappa_{1}\right)}^{2}W_{4}\left(1,q\right)\right),$$

*where*

$$\begin{array}{cc} \; & W_{4}\left(1,q\right)=\frac{q^{2}\left(q^{4}+q^{3}+q^{2}-q+1\right)}{q^{9}+3q^{8}+6q^{7}+9q^{6}+11q^{5}+11q^{4}+9q^{3}+6q^{2}+3q+1}\\ & W_{5}\left(1,q\right)=\frac{2q}{q^{3}+2q^{2}+2q+1}\\ & Z\left(1,q\right)=\max\left\{\left|{}_{\kappa_{1}}D_{q}K\left(\kappa_{1}\right)\right|,\left|{}_{\kappa_{1}}D_{q}K\left(\kappa_{2}\right)\right|\right\}. \end{array}$$

**Theorem** **10.** *If we suppose that all of the criteria of Lemma 5 are satisfied, then the resulting inequality, shows that ${\left|{}^{\kappa_{2}}D_{p,q}K\right|}^{\sigma }$ is a strongly convex functions on $\left[\kappa_{1},\kappa_{2}\right]$ with modulus χ≥1 for σ≥1, then*

(55) $$\begin{array}{c} \left|\frac{1}{p\left(\kappa_{2}-\kappa_{1}\right)}\int_{p\kappa_{1}+\left(1-p\right)\kappa_{2}}^{\kappa_{2}}K\left(x\right){\;}^{\kappa_{2}}d_{p,q}x-\frac{pK\left(\kappa_{1}\right)+qK\left(\kappa_{2}\right)}{{\left[2\right]}_{p,q}}\right|\leq q\left(\kappa_{2}-\kappa_{1}\right){\left[W_{5}\left(p,q\right)\right]}^{1-\frac{1}{\sigma }}\\ \times {\left[W_{1}\left(p,q\right){\left|{}_{\kappa_{1}}D_{p,q}K\left(\kappa_{1}\right)\right|}^{\sigma }+W_{2}\left(p,q\right){\left|{}_{\kappa_{1}}D_{p,q}K\left(\kappa_{2}\right)\right|}^{\sigma }-\chi {\left(\kappa_{1}-\kappa_{2}\right)}^{2}W_{4}\left(p,q\right)\right]}^{\frac{1}{\sigma }}, \end{array}$$

*where $W_{1}\left(p,q\right)$, $W_{2}\left(p,q\right)$, $W_{3}\left(p,q\right)$ and $W_{4}\left(p,q\right)$ are defined in Theorem 7.*

**Proof.**  The desired inequality (55) can be obtained by following the strategy applied in the proof of Theorem 7 and considering the Lemma 5. □

**Theorem** **11.** *If we suppose that all of the criteria of Lemma 5 are satisfied, then the resulting inequality shows that ${\left|{}^{\kappa_{2}}D_{p,q}K\right|}^{\sigma_{2}}$ is a strongly convex functions on $\left[\kappa_{1},\kappa_{2}\right]$ with modulus χ≥1 for $\frac{1}{\sigma_{1}}+\frac{1}{\sigma_{2}}=1,$ then*

(56) $$\begin{array}{c} \left|\frac{1}{p\left(\kappa_{2}-\kappa_{1}\right)}\int_{p\kappa_{1}+\left(1-p\right)\kappa_{2}}^{\kappa_{2}}K\left(x\right){\;}^{\kappa_{2}}d_{p,q}x-\frac{pK\left(\kappa_{1}\right)+qK\left(\kappa_{2}\right)}{{\left[2\right]}_{p,q}}\right|\leq q\left(\kappa_{2}-\kappa_{1}\right){\left[M\left(p,q\right)\right]}^{1-\frac{1}{\sigma_{1}}}\\ \times {\left(\frac{\left[{\left[2\right]}_{p,q}-1\right]{\left|{}_{\kappa_{1}}D_{p,q}K\left(\kappa_{1}\right)\right|}^{\sigma_{2}}+{\left|{}_{\kappa_{1}}D_{p,q}K\left(\kappa_{2}\right)\right|}^{\sigma_{2}}}{{\left[2\right]}_{p,q}}-\frac{\chi {\left(\kappa_{2}-\kappa_{1}\right)}^{2}\left[{\left[3\right]}_{p,q}-{\left[2\right]}_{p,q}\right]}{{\left[2\right]}_{p,q}{\left[3\right]}_{p,q}}\right)}^{\frac{1}{\sigma_{2}}}, \end{array}$$

*where $M\left(p,q\right)$ is defined in Theorem 8.*

**Proof.**  The desired inequality (56) can be obtained by following the strategy applied in the proof of Theorem 8 and considering the Lemma 5. □

**Theorem** **12.** *If we suppose that all of the criteria of Lemma 5 are satisfied, then the resulting inequality shows that ${\left|{}^{\kappa_{2}}D_{p,q}K\right|}^{\sigma }$ is a strongly quasi-convex functions on $\left[\kappa_{1},\kappa_{2}\right]$ with modulus χ≥1 for σ≥1, then*

(57) $$\begin{array}{c} \left|\frac{1}{p\left(\kappa_{2}-\kappa_{1}\right)}\int_{p\kappa_{1}+\left(1-p\right)\kappa_{2}}^{\kappa_{2}}K\left(x\right){\;}^{\kappa_{2}}d_{p,q}x-\frac{pK\left(\kappa_{1}\right)+qK\left(\kappa_{2}\right)}{{\left[2\right]}_{p,q}}\right|\\ \leq \frac{q\left(\kappa_{2}-\kappa_{1}\right)}{2^{1-\frac{1}{\sigma }}}{\left(W_{5}\left(p,q\right)\right)}^{1-\frac{1}{\sigma }}{\left(S\left(p,q\right)\left(W_{5}\left(p,q\right)\right)-\chi {\left(\kappa_{2}-\kappa_{1}\right)}^{2}W_{4}\left(p,q\right)\right)}^{\frac{1}{\sigma }}, \end{array}$$

*where*

$$S\left(p,q\right)=\max\left\{{\left|{}^{\kappa_{2}}D_{p,q}K\left(\kappa_{1}\right)\right|}^{\sigma },{\left|{}^{\kappa_{2}}D_{p,q}K\left(\kappa_{2}\right)\right|}^{\sigma }\right\},$$

*and $W_{4}\left(p,q\right)$, $W_{5}\left(p,q\right)$ are defined in Theorem 7.*

**Proof.**  The desired inequality (57) can be obtained by following the strategy applied in the proof of Theorem 9 and considering the Lemma 5. □

## 3. Examples

**Figure 1:**
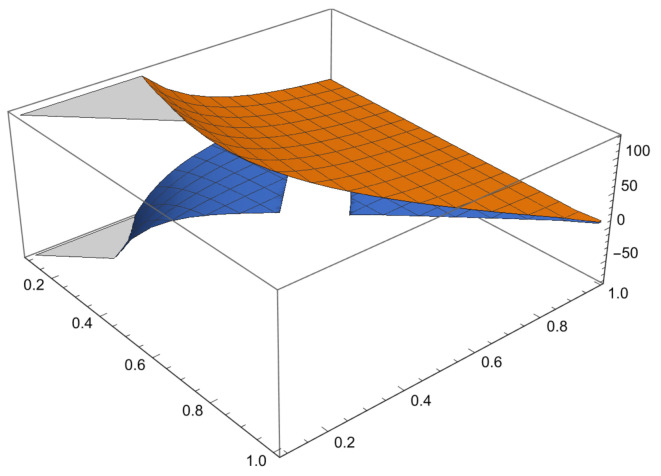
Comparison of error and error bound in (26).

**Figure 2:**
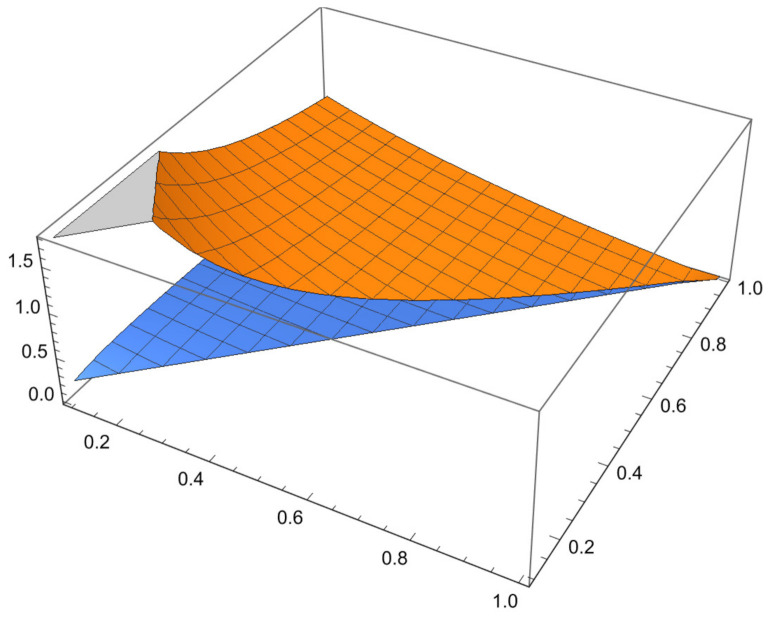
Comparison of error and error bound in (42).

**Figure 3:**
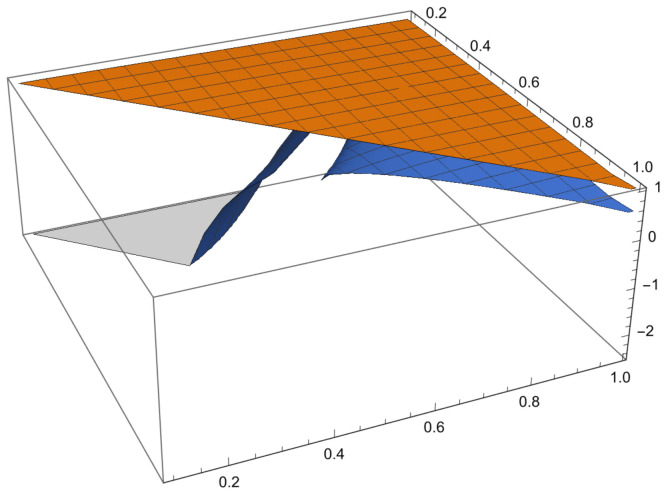
Comparison of error and error bound in (50).

Some examples are given to illustrate the investigated results and Figure 1 shown the comparison of error and error bound in (26), Figure 2 shown the comparison of error and error bound in (42)and Figure 3 shown the comparison of error and error bound in (50), respectively.

**Example** **1.** *Consider a function K:[0,3]→R by $K\left(x\right)=x^{2}$ with σ=4. Then, ${\left|{}_{0}D_{p,q}K\left(x\right)\right|}^{4}={\left|{}_{0}D_{p,q}x\right|}^{4}={\left[2\right]}_{p,q}^{4}x^{4}$ is a strongly convex functions on $\left[0,3\right].$ Then, K satisfies the conditions of Theorem 7 with 0<q<p≤1, so the left side of (26) becomes*

$$\begin{array}{cc} \; & \left|\frac{1}{p\left(\kappa_{2}-\kappa_{1}\right)}\int_{\kappa_{1}}^{\left(1-p\right)\kappa_{1}+p\kappa_{2}}K\left(x\right){\;}_{\kappa_{1}}d_{p,q}x-\frac{qK\left(\kappa_{1}\right)+pK\left(\kappa_{2}\right)}{{\left[2\right]}_{p,q}}\right|\\ \; & =\left|\frac{1}{p\left(3-0\right)}\int_{0}^{(1-p)0+3p}K\left(x\right){\;}_{\kappa_{1}}d_{p,q}x-\frac{qK\left(0\right)+pK\left(3\right)}{{\left[2\right]}_{p,q}}\right|\\ \; & =\left|\frac{27}{{\left[3\right]}_{p,q}}-\frac{9p}{{\left[2\right]}_{p,q}}\right|, \end{array}$$

*and the right side of (26) with χ=2 becomes*

(58) $$\begin{array}{c} q\left(\kappa_{2}-\kappa_{1}\right){\left[W_{5}\left(p,q\right)\right]}^{1-\frac{1}{\sigma }}{\left[W_{1}\left(p,q\right){\left|{}_{\kappa_{1}}D_{p,q}K\left(\kappa_{1}\right)\right|}^{\sigma }+W_{2}\left(p,q\right){\left|{}_{\kappa_{1}}D_{p,q}K\left(\kappa_{2}\right)\right|}^{\sigma }-\chi {\left(\kappa_{2}-\kappa_{1}\right)}^{2}W_{4}\left(p,q\right)\right]}^{\frac{1}{\sigma }}\\ =q\left(3-0\right){\left[W_{5}\left(p,q\right)\right]}^{1-\frac{1}{\sigma }}{\left[W_{1}\left(p,q\right){\left|{}_{\kappa_{1}}D_{p,q}K\left(0\right)\right|}^{\sigma }+W_{2}\left(p,q\right){\left|{}_{\kappa_{1}}D_{p,q}K\left(3\right)\right|}^{4}-\chi {\left(3-0\right)}^{2}W_{4}\left(p,q\right)\right]}^{\frac{1}{4}}\\ =3q{\left[\frac{2\left[{\left[2\right]}_{p,q}-1\right]}{{\left[2\right]}_{p,q}{\left[3\right]}_{p,q}}\right]}^{\frac{3}{4}}\left[81{\left[2\right]}_{p,q}^{2}\left[\frac{2{\left[2\right]}_{p,q}\left({\left[3\right]}_{p,q}-{\left[2\right]}_{p,q}\right)+{\left[4\right]}_{p,q}\left({\left[2\right]}_{p,q}^{2}-{\left[3\right]}_{p,q}\right)}{{\left[3\right]}_{p,q}{\left[4\right]}_{p,q}}\right]\right.\\ \left.-18\left[\frac{2{\left[2\right]}_{p,q}\left({\left[3\right]}_{p,q}-{\left[2\right]}_{p,q}\right)+{\left[4\right]}_{p,q}\left({\left[2\right]}_{p,q}^{2}-{\left[3\right]}_{p,q}\right)}{{\left[2\right]}_{p,q}^{2}{\left[3\right]}_{p,q}{\left[4\right]}_{p,q}}\right.\right.\\ {\left.\left.-\frac{2{\left[2\right]}_{p,q}\left[{\left[4\right]}_{p,q}-{\left[3\right]}_{p,q}\right]+{\left[5\right]}_{p,q}\left[{\left[2\right]}_{p,q}{\left[3\right]}_{p,q}-{\left[4\right]}_{p,q}\right]}{{\left[2\right]}_{p,q}{\left[3\right]}_{p,q}{\left[4\right]}_{p,q}{\left[5\right]}_{p,q}}\right]\right]}^{\frac{1}{4}}. \end{array}$$

**Example** **2.** *Consider a function K:[0,1]→R by K(x)=1−x with $\sigma_{1}=\sigma_{2}=2$. Then, ${\left|{}_{0}D_{p,q}K\left(x\right)\right|}^{4}={\left|{}_{0}D_{p,q}\left(1-x\right)\right|}^{4}=1$ is a strongly convex functions on $\left[0,1\right].$ Then, K satisfies the conditions of Theorem 8 with 0<q<p≤1, so the left side of (42) becomes*

$$\begin{array}{cc} \; & \left|\frac{1}{p\left(\kappa_{2}-\kappa_{1}\right)}\int_{\kappa_{1}}^{\left(1-p\right)\kappa_{1}+p\kappa_{2}}K{\left(x\right)}_{\kappa_{1}}d_{p,q}x-\frac{qK\left(\kappa_{1}\right)+pK\left(\kappa_{2}\right)}{{\left[2\right]}_{p,q}}\right|\\ \; & =\left|\frac{1}{p\left(1-0\right)}\int_{0}^{(1-p)0+1p}K\left(x\right){\;}_{\kappa_{1}}d_{p,q}x-\frac{qK\left(0\right)+pK\left(1\right)}{{\left[2\right]}_{p,q}}\right|\\ \; & =\left|\frac{{\left[2\right]}_{p,q}-1}{{\left[2\right]}_{p,q}}-\frac{q}{{\left[2\right]}_{p,q}}\right|, \end{array}$$

*and the right side of (42) with χ=3 becomes*

(59) $$\begin{array}{c} q\left(\kappa_{2}-\kappa_{1}\right){\left[M\left(p,q\right)\right]}^{1-\frac{1}{\sigma_{1}}}{\left(\frac{\left[{\left[2\right]}_{p,q}-1\right]{\left|{}_{\kappa_{1}}D_{p,q}K\left(\kappa_{1}\right)\right|}^{\sigma_{2}}+{\left|{}_{\kappa_{1}}D_{p,q}K\left(\kappa_{2}\right)\right|}^{\sigma_{2}}}{{\left[2\right]}_{p,q}}-\frac{\chi {\left(\kappa_{2}-\kappa_{1}\right)}^{2}\left[{\left[3\right]}_{p,q}-{\left[2\right]}_{p,q}\right]}{{\left[2\right]}_{p,q}{\left[3\right]}_{p,q}}\right)}^{\frac{1}{\sigma_{2}}}\\ =q{\left[M\left(p,q\right)\right]}^{\frac{1}{2}}{\left(\frac{\left[{\left[2\right]}_{p,q}-1\right]}{{\left[2\right]}_{p,q}}-\frac{3\left[{\left[3\right]}_{p,q}-{\left[2\right]}_{p,q}\right]}{{\left[2\right]}_{p,q}{\left[3\right]}_{p,q}}\right)}^{\frac{1}{2}}, \end{array}$$

*where $M\left(p,q\right)$ is defined in Theorem 8.*

*The series above can be shown to be convergent. The graph below shows that the LHS is less than or equal to the RHS. Therefore, the inequality (42) is valid for the particular choice of the function K:[0,1]→R defined by K(x)=1−x with $\sigma_{1}=\sigma_{2}=2$ and ${\left|{}_{0}D_{p,q}K\left(x\right)\right|}^{4}={\left|{}_{0}D_{p,q}\left(1-x\right)\right|}^{4}=1$, which is a strongly convex functions on $\left[0,1\right]$*

**Example** **3.** *Consider a function K:[0,1]→R by $K\left(x\right)=\frac{1}{16}x^{2}$ with σ=3. Then, ${\left|{}_{0}D_{p,q}K\left(x\right)\right|}^{3}=\frac{1}{16}{\left|\left(p+q\right)\left(x+1\right)-2\right|}^{3}$ is a strongly quasi-convex functions on $\left[-1,1\right]$. Then K satisfies the conditions of Theorem 9 with 0<q<p≤1, so the left side of (50) becomes*

$$\begin{array}{cc} \; & \left|\frac{1}{p\left(\kappa_{2}-\kappa_{1}\right)}\int_{\kappa_{1}}^{\left(1-p\right)\kappa_{1}+p\kappa_{2}}K{\left(x\right)}_{\kappa_{1}}d_{p,q}x-\frac{qK\left(\kappa_{1}\right)+pK\left(\kappa_{2}\right)}{{\left[2\right]}_{p,q}}\right|\\ \; & =\left|\frac{1}{2p}\int_{-1}^{2p-1}x^{2}{\;}_{-1}d_{p,q}x-\frac{qK\left(-1\right)+pK\left(1\right)}{{\left[2\right]}_{p,q}}\right|\\ \; & =\left|\frac{1}{32p}\left[\frac{8p^{3}}{{\left[3\right]}_{p,q}}-\frac{8p^{2}}{{\left[2\right]}_{p,q}}+2p\right]-1\right|, \end{array}$$

*and the right side of (50) with $\chi =\frac{1}{20}$ becomes*

(60) $$\begin{array}{c} q\left(\kappa_{2}-\kappa_{1}\right){\left[W_{5}\left(p,q\right)\right]}^{1-\frac{1}{\sigma }}{\left[Z\left(p,q\right)W_{5}\left(p,q\right)-\chi {\left(\kappa_{2}-\kappa_{1}\right)}^{2}W_{4}\left(p,q\right)\right]}^{\frac{1}{\sigma }}\\ =2q{\left[\frac{2\left[{\left[2\right]}_{p,q}-1\right]}{{\left[2\right]}_{p,q}{\left[3\right]}_{p,q}}\right]}^{\frac{2}{3}}\left[\max\left\{\frac{1}{2},\frac{1}{2}{\left|{\left[2\right]}_{p,q}-1\right|}^{3}\right\}\left[\frac{2\left[{\left[2\right]}_{p,q}-1\right]}{{\left[2\right]}_{p,q}{\left[3\right]}_{p,q}}\right]\right.\\ \left.-\frac{1}{5}\left[\frac{2{\left[2\right]}_{p,q}\left({\left[3\right]}_{p,q}-{\left[2\right]}_{p,q}\right)+{\left[4\right]}_{p,q}\left({\left[2\right]}_{p,q}^{2}-{\left[3\right]}_{p,q}\right)}{{\left[2\right]}_{p,q}^{2}{\left[3\right]}_{p,q}{\left[4\right]}_{p,q}}\right.\right.\\ {\left.\left.-\frac{2{\left[2\right]}_{p,q}\left[{\left[4\right]}_{p,q}-{\left[3\right]}_{p,q}\right]+{\left[5\right]}_{p,q}\left[{\left[2\right]}_{p,q}{\left[3\right]}_{p,q}-{\left[4\right]}_{p,q}\right]}{{\left[2\right]}_{p,q}{\left[3\right]}_{p,q}{\left[4\right]}_{p,q}{\left[5\right]}_{p,q}}\right]\right]}^{\frac{1}{3}}. \end{array}$$

*From the graph below, it is obvious that the LHS is less than or equal to the RHS. Therefore, the inequality (50) is valid for every strongly quasi-convex functions.*

## 4. Conclusions

Convex functions are represented in terms of different inequalities. Many of the well-known inequalities are consequences of convex functions. Strong convexity is a strengthening of the notion of convexity; some properties of strongly convex functions are just stronger versions of known properties of convex functions. In this research, we identified new results that are used to calculate $\left(p,q\right){}_{\kappa_{1}}$ and $\left(p,q\right){}^{\kappa_{2}}$—trapezoidal integral-type inequalities through strongly convex and quasi-convex functions. Furthermore, some examples were presented to illustrate the outcome of the research.

## Data Availability

Not applicable.
